# A randomized cross-over trial investigating differences in 24-h personal air and skin temperatures using wearable sensors between two climatologically contrasting settings

**DOI:** 10.1038/s41598-021-01180-y

**Published:** 2021-11-10

**Authors:** Andria Constantinou, Stavros Oikonomou, Corina Konstantinou, Konstantinos C. Makris

**Affiliations:** Cyprus International Institute for Environmental and Public Health, Cyprus University of Technology, Limassol, Cyprus

**Keywords:** Sensors and probes, Risk factors

## Abstract

The influence of elevated air temperatures recorded in various urban microenvironments in adversely impacting biologically relevant disease end points has not yet been extensively tackled. This study is a post hoc analysis of the TEMP pilot trial, a randomized 2 × 2 cross-over trial that examined changes in metabolic and stress hormonal profiles of healthy adults in two settings (urban vs. rural) with distinctly different climatological characteristics during the Mediterranean summer. This analysis aimed to study the association between the 24-h personal air or skin temperature sensor measurements and the diary-based location type (indoors vs. outdoors) in urban (seaside) vs. rural (higher in altitude) microenvironments. Out of 41 eligible participants, a total of 37 participants were included in this *post-hoc* TEMP trial analysis. Wearable sensors recorded personal air temperature, skin temperature, and activity (as a surrogate marker of physical activity) in each setting, while a time-stamped personal diary recorded the types of indoor or outdoor activities. Temperature peaks during the 24-h sampling period were detected using a peak finding algorithm. Mixed effect logistic regression models were fitted for the odds of participant location (being indoors vs. outdoors) as a function of setting (urban vs. rural) and sensor-based personal temperature data (either raw temperature values or number of temperature peaks). During the study period (July–end of September), median [interquartile range, IQR] personal air temperature in the rural (higher altitude) settings was 1.5 °C lower than that in the urban settings (27.1 °C [25.4, 29.2] vs. 28.6 °C [27.1, 30.5], *p* < 0.001), being consistent with the Mediterranean climate. Median [IQR] personal air temperature in indoor (micro)environments was lower than those in outdoors (28.0 °C [26.4, 30.3] vs 28.5 °C [26.8, 30.7], *p* < 0.001). However, median [IQR] skin temperature was higher in indoor (micro)environments vs. outdoors (34.8 °C [34.0, 35.6] and 33.9 °C [32.9, 34.8], *p* < 0.001) and the number of both personal air and skin temperature peaks was higher indoors compared to outdoors (median [IQR] 3.0 [2.0,4.0] vs 1.0 [1.0,1.3], *p* < 0.007, for the skin sensors). A significant association between the number of temperature peaks and indoor location types was observed with either the personal air sensor (OR 3.1; 95% CI 1.2–8.2; *p* = 0.02) or the skin sensor (OR 3.7; 95% CI 1.4–9.9; *p* = 0.01), suggesting higher number of indoor air temperature fluctuations. Amidst the global climate crisis, more population health studies or personalized medicine approaches that utilize continuous tracking of individual-level air/skin temperatures in both indoor/outdoor locations would be warranted, if we were to better characterize the disease phenotype in response to climate change manifestations.

## Introduction

The global climate crisis has highlighted the importance of key proximate drives of planetary health, such as elevated air temperatures that may adversely impact human health^1^. Changes in air temperature directly affect the body's physiological functions^2,3^, such as the ability to regulate its internal temperature^4^. Literature has linked increased ambient temperatures with premature mortality in Europe^5,6^, Australia^7–9^, the United States^10–12^, and Korea^13^. The association between elevated ambient temperatures and the burden of disease has been highlighted for a series of renal, cardiovascular, respiratory and infectious disease outcomes^14–17^.

Monitoring the magnitude and variation of air temperature in various microenvironments, such as inside a household, or inside a car/bus, or being in a park is key to providing more accurate exposure estimates towards the characterization of the temperature-disease outcome continuum^18,19^. Wearable sensors offer the option of continuous individual-level exposure assessment for epidemiological studies^20^. Wearable temperature sensors go beyond the classical approach of collecting ambient air temperature data from meteorological stations for use in epidemiological studies of infectious diseases, as in COVID-19^21,22^ or in studies of cardiovascular disease outcomes^23^. The use of such stationary-based temperature data may not capture well the diurnal variation in within-subject ambient temperatures, since individuals spend a limited amount of time in close proximity to meteorological stations and tend to spend varying time in different indoor microenvironments during the day with variable indoor air temperature profiling, leading often to exposure misclassification bias^24^.

Wearable sensors of temperature measurements in either the personal air, or skin, or body core temperature have already been used in studies of environmental epidemiology. For example, low-cost wearable sensors (attached to participant’s lapel, or shoes, or backpack) were used for depicting differences between personal air temperature and temperature based on weather stations^25^. The SenseIO system provides fine-grained detection using measurements of sensor-rich smartphones (e.g., cellular, Wi-Fi, accelerometer, proximity, light-, and time-clock) to infer automatically the ambient environment type (i.e., rural, urban, indoor and complex places)^26^. A combination of built-in smartphone sensors, portable monitors, and geographic data comprise of the Expoapp system in providing a suite of dynamic environmental exposure measurements^27^. Other sensors attempt to accurately classify participant location using indoor positioning methods based on the Received Signal Strength (RSS) fingerprint or a cluster principal component analysis-based indoor positioning algorithm^28,29^. Wireless temperature skin sensors have been used to test the hypothesis whether the temporal association between personal air temperature and blood pressure was mediated via skin temperature, and whether this relationship was season-dependent^30^. Human activity within near extreme temperature environments has been monitored with the aid of ingestible thermometer sensors that track core (gastro-intestinal) body temperature, e.g., in elite alpine skiers^31^.

The influence of elevated air temperatures measured in various urban microenvironments, particularly those indoors (household rooms, working place, gym, restaurant, etc.), on biologically relevant disease end points has not yet been extensively tackled. Wearable sensors represent a novel, non-invasive and cost-effective means of monitoring personal exposures to a suite of environmental agents, including temperature variation^32^. Towards this, the pilot TEMP trial was conducted in real-life conditions, moving away from studies focused on well-controlled temperature indoor settings that typically expose participants to a discrete set of temperatures for a pre-specified period. The TEMP trial showed that a short-term stay (5–7 days) in climatologically cooler areas during summer (e.g., in rural) improved the profile of a metabolic hormone (leptin) for non-obese healthy adults who permanently reside in urban areas of a Mediterranean country^33^.

Thus, the objectives of this post hoc TEMP trial analysis were to: i) characterize the diurnal variation in the sensor-based personal air- and skin-temperatures, including the number of temperature peaks in each setting (urban vs. rural) and location type (indoors vs. outdoors), and ii) study the association between the various sensor-based individual 24-h temperature measurements and the diary-based location type (indoors) in both study settings.

## Methods

### Study design and population

This study is a post hoc analysis of temperature sensors and diary data obtained from the TEMP pilot trial^33^. The TEMP randomized 2 × 2 cross-over trial examined changes in metabolic and stress hormonal profiles of healthy, non-obese adults (n = 41) in two study settings with distinctly different climatological characteristics: i) urban setting (range: < 100–250 m altitude) vs. higher in altitude (range: 650–1200 m, mean ± SD: 881 ± 200 m) rural setting. The rural setting was defined as a mountainous area (~ 260km^2^) located in short driving distance ~ 1-h drive from the main urban centres of Cyprus with distinctly cooler meteorological characteristics than those of the urban centers. The geographical cluster of the study’s rural communities was relatively small with the longest and the shortest distance between the rural communities of the study being about 1-h of driving (61 km) and 10-min of driving (3.8 km), respectively.

Random allocation of the intervention was made a priori to two groups, based on the study protocol. The permanent residence of all participants was located in the urban setting. Group A included volunteers spending time in their urban setting and then moving to the rural setting for at least 5 days (n = 12); Group B included individuals who initially stayed in the rural setting for at least 5 days and then returned to their urban residence (n = 29). During recruitment, 8 participants opted to change group, resulting to unequal group sizes. Eligible participants for this post hoc analysis were those having collected at least one sensor dataset (either skin or personal air sensor) in at least one setting (urban or rural) with the corresponding 24-h diary data.

The trial and its post hoc protocols were approved by the Cyprus National Bioethics Committee (ΕΕΒΚ/ΕΠ/2018/30). The trial was registered in the US (ClinicalTrials.gov Identifier: NCT03625817) and the participants signed a written informed consent prior to study initiation. Αll methods were carried out in accordance with relevant guidelines and regulations.

### Data collection

Data was collected in Cyprus from July until end of September, 2018^33^. From the 41 eligible participants that were recruited for TEMP trial analysis, 2 withdrew from the study and another 2 declined participation; a total of 37 participants were included in the *post-hoc* analysis. During the two sampling days (one sampling day per setting), the participants noted down their diurnal activities in a diary and recorded physical parameters such as activity, personal air and skin temperature by wearing personal temperature sensors (used as a tag and attached on skin, respectively).

#### Diary data

A self-reported circa 24-h diary was completed by each participant on the sampling day (e.g., breakfast, lunch, entrance/exit from buildings, sleep/wake cycle and indoor/outdoor physical activities, etc.), starting from 04:00 on sampling day until ~ 12:00 noon on the day following the sampling day. The diary consisted of 30-min interval periods so that activities’ time stamp and duration would be easier for participants to record.

#### Sensors

Wearable sensors (e-TACT, BodyCAP Medical, France) were used for the personal air and skin temperature measurements, as well as activity tracking (as a surrogate marker of physical activity). Two sensors were given per participant; one was worn as a tag on the participants’ chest area for the personal air measurement and another one, was attached directly to their left armpit, to measure the skin temperature, on the night before the sampling day and disconnected on the day following the sampling day. Data entries used in the analysis spanned from 05:00 on the sampling day to 04:59 of the next day.

Activity data was collected using the skin sensor. The activity sensor tracking mode used the time above threshold (TAT) method typically used for physical activity measurements^34^, using temperature measurement period (1 min), sampling frequency (50 Hz), actimetry period (1 min), accelerometer sensitivity threshold to consider an activity as important or not (0.1 g), and the measurement range (2G). The high sampling frequency was warranted to ensure capturing most, if not all episodic or peak types of exposures encountered during the 24-h period of measurements with the skin or personal air temperature sensors.

### Data pre-processing

Raw diary data digitization was executed in Excel in English (original diaries were written in Greek language), while an expanded version was then created presenting activities per minute from 05:00 to 04:59, using the built-in rep() function in R. The Multinational Time Use Study (MTUS) guidance was used to deal with missing values^35^. Specifically, 30–60 min gap between two different locations were filled with “unreported transport” and gaps between “sleep” and “wake up” activities, were filled with “sleep”. In addition, gaps between “being at work” and driving (where driving, based on the diary of each participant, refers to the exit from their workplace and car usage) were filled with ‘being at work’.

After the MTUS imputation process, self-reported activity diary had 37% and 30% missing values in rural and urban settings, respectively (Table S1). Activities were included in sub-groups based on their type (e.g. activities such as ‘watching TV’ and ‘Housekeeping’ were grouped to ‘Inside house’) and then included in three wider groups according to their location; where location was clearly reported in the diary, activities were included as “indoor activities” or “outdoor activities”. Any other activities were included in the “unspecified location” group and were excluded from the analysis, as well as missing values. The percentage of participants’ time spent in each location during the 24-h period, including missing values was also calculated (Table S1).

We assumed non-compliance with properly using the skin sensor, if skin temperature values were < 30 °C during the 24-h period and > 99% of activity levels were equal to zero. Based on the literature, human skin can adapt temperatures between 29 and 37 °C in normal conditions^36^ with the minimum skin temperatures measured in distal body parts being > 30 °C in ambient air temperatures of 24–30 °C^31,37,38^. As such, skin sensor temperatures ≥ 30 °C were included in the final data analysis because we assumed that the participant was not wearing the sensor, if skin temperature data were < 30 °C. Diary and temperature data was merged according to the sensor type (air sensors dataset (ASD) and skin sensors dataset (SSD)) and smoothed using a local polynomial regression (LOESS) model^39^ using a smoothing span of 0.1^40^.

Temperature peaks during a 24-h sampling period were detected using the Findpeaks function, a general function that determines local peaks^41^, keeping the same optimized temperature threshold value of 0.2 °C for both sensor types (skin and personal air) (rest input parameters kept as default, see Supplementary Material). Using a range of 0.1–0.7 temperature threshold values, we observed that lower thresholds were able to detect more temperature peaks (Supplementary Material); as such, we chose the threshold value of 0.2 as the one being next to the lowest extreme value of 0.1.

The outcome was the participant location type (indoors or outdoors) that was classified using the 24-h diary data. A few datasets were created for the analysis of outcome. Diary data (indoor/outdoor entries) were merged with ASD and SSD by time, creating the air sensors including location dataset (ASLD) and skin sensors including location dataset (SSLD), respectively. Using findpeaks() function and ASD/SSD (including diary data), two additional datasets were created; peaks air sensors’ dataset including location (PASL) and peaks skin sensors’ dataset including location (PSSL), including only data entries with an indoor/outdoor activity group. PASL and PSSL consisted of the number of peaks and temperature values, indoor/outdoor location and activity (only for skin sensors’ data) for each peak. Participants with no peaks or having a peak time mismatch with an activity reported in the diary (indoors or outdoors) were not included in the datasets (PASL or PSSL).

### Statistical analysis

The baseline characteristics were presented overall, and by study group. Frequencies and percentages were used for the description of categorical variables and means (standard deviations) or medians (interquartile ranges, IQR) for the continuous variables. Non-normally distributed continuous variables (e.g., temperature) were compared using the nonparametric Wilcoxon test, whereas, normally distributed continuous variables were compared using the t-test. For the categorical variables, Fisher’s Exact test was used.

Mixed effect logistic regression models were fitted separately for each sensor type for the odds of participant location (being indoors vs. outdoors) as a function of the setting (urban vs. rural), raw sensor temperature data and activity (any vs. no activity; only in the models of skin sensors data). Similar models, without activity, were fitted including the interaction term between temperature and setting. Raw sensor temperature values were log-transformed, scaled and centered (z-score transformation). Activity was used as a binary variable due to a large number of zero activity values. Using the above models, the association between the location (indoors vs. outdoors) and the sensor-based diurnal temperature profiling was examined. Another set of models were fitted for the odds of participant location (being indoors vs. outdoors) as a function of the setting and number of temperature peaks.

Since the interaction term between temperature and setting was significant (*p* < 0.001) for both personal air and skin temperature-based models, mixed effect models stratified by setting were fitted (per sensor type). All models included participant-level random intercept with unstructured covariance matrix and had location as a binary outcome with outdoors location being the reference group. Rural setting was the reference group for the setting variable. Statistical tests and confidence intervals were two-sided with the statistical significance level set at 5% and 95% confidence intervals were presented. All analyses were performed in R (v. 4.0.2) and RStudio (v. 1.3.1073). The input data, scripts, and output are available in the Supplementary Material.

## Results

### Population characteristics

**Figure 1:**
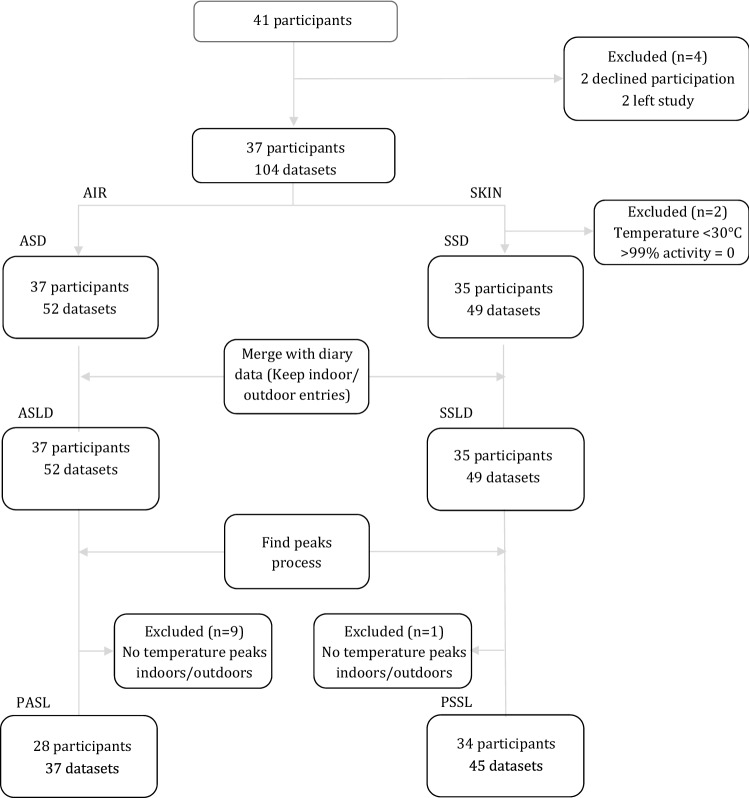
Flow chart of participants and datasets included in the analysis per sensor type.

Out of 41 subjects initially recruited and agreed to participate in the TEMP trial, 2 declined participation following the house visit (Fig. 1) and 2 did not collect any sensor data in any of the settings, while all of the remaining participants had the corresponding 24-h diary completed. A total of 37 participants (38% males, 41.6 ± 10.5 years old, BMI 24.8 ± 3.6 kg/m^2^) were included in this post hoc main analysis; 26 of them were allocated in Group B (first rural and then crossed over to the urban setting) and the rest in Group A (first urban and then crossed over to the rural setting). At baseline, high educational attainment was noted, with the majority holding at least a university/college degree (73%) and most participants were non-smokers (68%) with intermediate chronotype (69%), spending about 6-h per day on electronic screens. About half of them reported not regularly exercising (51%) and 38% reported never/rarely consuming alcohol (Table 1).

**Table 1:** Baseline characteristics of the study population overall and by group (Group A: first urban, Group B: first rural).

	Overall	Group A	Group B	*p* value*
N	37	11	26	
Age (mean, SD)	41.58 (10.45)	39.8 (6.83)	42.27 (11.58)	0.533
Sex = male (n, %)	14 (37.8)	2 (18.2)	12 (46.2)	0.150
BMI (mean kg/m^2^ (SD))	24.84 (3.62)	25.06 (4.46)	24.75 (3.29)	0.818
BMI (n, %)				0.459
Underweight	1 (2.7)	1 (9.1)	0 (0.0)	
Normal weight	21 (56.8)	5 (45.5)	16 (61.5)	
Overweight	12 (32.4)	4 (36.4)	8 (30.8)	
Obese	3 (8.1)	1 (9.1)	2 (7.7)	
Education (n, %)				0.189
Secondary	10 (27.0)	3 (27.3)	7 (26.9)	
University/college	13 (35.1)	6 (54.5)	7 (26.9)	
Master/PhD	14 (37.8)	2 (18.2)	12 (46.2)	
Chronotype^ (n, %)				0.009
Early	6 (23.1)	5 (50.0)	1 (6.2)	
Intermediate	18 (69.2)	4 (40.0)	14 (87.5)	
Late	2 (7.7)	1 (10.0)	1 (6.2)	
Smoking status (n, %)				0.867
Smoker	7 (18.9)	2 (18.2)	5 (19.2)	
Non-smoker	25 (67.6)	7 (63.6)	18 (69.2)	
Former smoker	5 (13.5)	2 (18.2)	3 (11.5)	
Alcohol consumption (n, %)				0.893
Weekly	16 (43.2)	4 (36.4)	12 (46.2)	
Monthly	7 (18.9)	2 (18.2)	5 (19.2)	
Rarely/never	14 (37.8)	5 (45.5)	9 (34.6)	
Physical exercise = No (n, %)	19 (51.4)	8 (72.7)	11 (42.3)	0.151
Screen time (hours/day) (mean, SD)	5.93 (3.76)	6.82 (3.49)	5.56 (3.87)	0.358
Days at rural setting (mean, SD)	7.05 (2.49)	6.73 (1.10)	7.19 (2.90)	0.611
Wash-out period (days) (mean, SD)	14.92 (9.05)	14.55 (9.28)	15.08 (9.13)	0.873

*The above were tested for differences between the two groups by Fisher’s Exact test for categorical variables and t-test for normally distributed continuous variables.

^the chronotype categories cut-offs are: early (< 3:00), intermediate (3:00–5:00) and late (> 5:00)^42^.

### Urban and rural temperature profile variation

Participants (n = 37) had data from the wearable skin and personal air sensors in either the urban or rural settings, or both. A total of 19 participants had at least one sensor dataset in both settings, 17 participants had at least one sensor dataset only in urban setting and 1 participant had at least one sensor dataset only in rural setting. Two participants were excluded from the skin sensor-related analysis, because all of their temperature entries were < 30 °C and more than 99% of their activity levels were equal to zero. Thus, 35 participants and 49 datasets, were included in SSD, while 37 participants and 52 datasets were included in ASD (none excluded). From the 37 participants in ASLD, peaks in data entries with an indoor/outdoor activity group were detected only in datasets of the 28. From the 35 participants in SSLD, 1 participant was not included for the same reason. Thus, the final PASL consisted of 28 participants with at least one peak in either indoor or outdoor location, whereas 34 participants were included in the final PSSL.

Skin temperature sensors generated a large dataset (20,301–44,967 data counts). Median [IQR] skin temperature was 34.64 °C [33.88, 35.39] and 34.61 °C [33.82, 35.34] in urban and rural settings, respectively, and the difference between the median skin temperatures across the two settings was significant (*p* < 0.001) (Table 2). Personal air temperature sensors produced diurnal data points in the range of 23,040–51,610 data counts. During the study period (July–end of September), median [IQR] personal air temperature in rural (higher altitude) settings was 1.5 °C lower (*p* < 0.001) than those in the urban settings (27.1 °C [25.4, 29.2] vs. 28.6 °C [27.1, 30.5]) (Table 2). The 24-h activity tracking showed that the median participants’ activity was lower in the rural setting compared with the urban setting (median [IQR] 0 [0,76] vs. 1 [0,67], respectively, *p* < 0.001*;* Table 2), being consistent with studies pointing out that urban dwellers tend to be more active in urban settings than when they temporarily moved to rural settings^43,44^. A total of 118 and 163 peaks were detected from the participants’ 24-h personal air and skin temperature sensor datasets, respectively. The number of both personal air peaks and skin temperature peaks did not significantly (*p* > 0.24 for skin temperature peaks) differ between the urban and rural settings (median [IQR] 2.5 [2, 4] vs. 2.0 [1, 3] for the skin peaks) (Table 2).

**Table 2:** Wearable sensor-based air and skin temperature diurnal variation, number of peaks and activity measurements stratified by setting (urban or rural).

	Urban		Rural		*p* value*
	n	Median [IQR]	n	Median [IQR]	
Personal air temperature (C°)	51,610	28.62 [27.14, 30.51]	23,040	27.05 [25.36, 29.19]	< 0.001
Skin temperature (C°)	44,967	34.64 [33.88, 35.39]	20,301	34.61 [33.82, 35.34]	< 0.001
Activity^1^	44,967	1.00 [0.00, 67.00]	20,301	0.00 [0.00, 76.00]	0.001
Personal air peaks temperature (C°)	92	31.34 [29.59, 32.79]	26	28.76 [26.90, 30.72]	0.001
Skin peaks temperature (C°)	109	35.25 [34.79, 35.89]	54	35.36 [34.54, 35.80]	0.430
# Personal air peaks	35	2.00 [1.00, 4.00]	17	1.00 [1.00, 2.00]	0.113
# Skin peaks	36	3.00 [2.00, 4.00]	21	2.00 [1.00, 4.00]	0.236

^1^Activity based on skin temperature sensor.

*Based on the Wilcoxon rank sum test.

### Indoors vs. outdoors diurnal variation in the sensor-based personal air- and skin-temperatures

During the study period (July–end of September), personal air temperature was significantly different (*p* < 0.001) between indoor and outdoor (micro)environments with lower median [IQR] personal air temperature in the indoor location (28.0 °C [26.4, 30.3] vs 28.5 °C [26.8, 30.7], respectively) (Table 3). At the same time, median skin temperature [IQR] was higher in indoor (micro)environments vs. outdoor (34.81 °C [34.04, 35.57] and 33.92 °C [32.87, 34.77]) (*p* < 0.001) (Table 3). Also, the number of both personal air peaks and skin temperature peaks was significantly (*p* < 0.001) different between indoors and outdoors with higher median [IQR] number of peaks in indoor (micro)environments compared to outdoor (e.g., median [IQR]: 3.0 [2.0, 4.0] vs 1.0 [1.0, 1.25] for the skin sensors) (Table 3). Among all the wider groups’ activities (indoors, outdoors and unspecified location), participants spent more than the half of their 24-h time indoors (53%) (Table S1). The entry of ‘sleep’ activity during the 24-h period in both settings (~ 32% in rural and ~ 30% in urban settings) contributed the most time among all activities during the 24-h time period (Table S4). The higher percentage of being outdoors during the 24-h period in the rural setting (5.7% vs 0.7% in urban setting) could be explained by the greater amount of free time (working place was located in urban settings for all, ~ 16% of the 24-h time vs. ~ 1% devoted to work activities in rural setting) (Table S4).

**Table 3:** Wearable sensor-based air and skin temperature diurnal variation, number of peaks and activity measurements stratified by location (indoors or outdoors).

	Indoors		Outdoors		*p* value*
	n	Median [IQR]	n	Median [IQR]	
Personal air temperature (C°)	39,696	27.95 [26.37, 30.27]	3176	28.52 [26.79, 30.65]	< 0.001
Skin temperature (C°)	35,653	34.81 [34.04, 35.57]	3986	33.92 [32.87, 34.77]	< 0.001
Activity^1^	35,653	0.00 [0.00, 7.00]	3986	70.50 [1.00, 267.00]	< 0.001
Personal air peaks temperature (C°)	97	30.98 [28.67, 32.78]	21	30.13 [27.94, 32.17]	0.167
Skin peaks temperature (C°)	146	35.29 [34.76, 35.95]	17	34.91 [34.59, 35.52]	0.104
# Personal air peaks	35	2.00 [1.00, 4.00]	17	1.00 [1.00, 1.00]	0.001
# Skin peaks	45	3.00 [2.00, 4.00]	12	1.00 [1.00, 1.25]	< 0.001

^1^Activity based on skin temperature sensor.

*Based on the Wilcoxon rank sum test.

### Association between the location (indoors vs. outdoors) and the sensor-based diurnal temperature profiling

Being indoors during a Mediterranean summer was associated with a few important covariates. Living in an urban setting during summer was associated with a significantly higher odds ratio (OR) of being located indoors rather than outdoors during an average 24-h time period (OR 3.54; 95% CI 3.20–3.92; *p* < 0.001, based on the personal air sensor data; OR 3.08; 95% CI 2.80–3.39; *p* < 0.001 based on the skin sensor data) (Table 4). Being indoors was twice as likely to occur with an increase of 1 standard deviation (SD) of skin sensor-based temperatures (OR 2.07; 95% CI 1.97–2.16; *p* < 0.001), while being outdoors was associated with higher (1 SD increase) personal air temperatures (OR 0.66; 95% CI 0.63–0.70; *p* < 0.001) (Table 4).

**Table 4:** Mixed effect models of participant location (indoors vs outdoors) as a function of the setting, temperature and activity groups using either the personal air or skin crude temperature sensor data.

	Location (indoors) (personal air temperature data)		*p* value	Location (indoors) (skin temperature data)		*p* value
	OR	95% CI		OR	95% CI	
Setting (urban)	3.541	3.200–3.918	** < 0.001**	3.079	2.795–3.392	** < 0.001**
Temperature	0.664	0.632–0.699	** < 0.001**	2.065	1.972–2.162	** < 0.001**
Activity (any activity)				0.211	0.193–0.230	** < 0.001**
Within participant variance	3.29			3.29		
Between participant variance	9.30			9.54		
ICC	0.74			0.74		
Observations	42,872			39,639		

Statistically significant associations (*p*-value < 0.05) are shown in bold.

Participant activity (yes vs no activity) was significantly associated with outdoors (micro)environments (OR 0.21; 95% CI 0.19–0.23; *p* < 0.001) (Table 4). A significant interaction (*p* < 0.001) between temperature and setting (urban/rural) was found in both personal air and skin sensor temperature data (Table S2); for personal air sensors, the urban model showed: OR 0.42; 95% CI 0.38–0.46; *p* < 0.001 and the rural model: OR 0.52; 95% CI 0.48–0.56; *p* < 0.001; while for skin sensors data, the urban model showed: OR 1.85; 95% CI 1.71–1.99; *p* < 0.001 and the rural model: OR 3.13; 95% CI 2.93–3.33; *p* < 0.001 (Table S3). A significant association between the number of temperature peaks and indoors location was observed with either the personal air sensor (OR 3.42; 95% CI 1.24–9.39; *p* = 0.017) or the skin sensor (OR 3.62; 95% CI 1.38–9.49; *p* = 0.009) (Table 5).

**Table 5:** Mixed effect models of participant location as a function of the setting, number of temperature peaks using either the personal air-, or skin-based number of temperature peaks from the sensor data.

	Location (indoors) (personal air temperature data)		*p* value	Location (indoors) (skin temperature data)		*p* value
	OR	95% CI		OR	95% CI	
Setting (Urban)	1.157	0.306–4.376	0.830	2.379	0.522–10.831	0.263
# temperature peaks	3.418	1.244–9.390	**0.017**	3.617	1.379–9.486	**0.009**
Within participant variance	3.29	3.29				
Between participant variance	0.00	0.00				
Observations	52	57				

Statistically significant associations (*p*-value < 0.05) are shown in bold.

## Discussion

Using data from personal air and skin temperature sensors, as well as the participant diaries from the main trial^33^, this post hoc trial investigated the association between the 24-h diurnal variation of wearable temperature sensor data and the odds of being located in any indoor (vs. outdoors) (micro)environment. Using wearable sensors, adult volunteers collected temperature data at both urban and rural (micro)environments (cross-over design) during the summer period of a Mediterranean country (Cyprus). Both raw temperature data and the estimated number of temperature peaks during a 24-h sampling day in each setting (urban and rural) were used.

Overall, lower median skin and personal air temperature values and number of temperature peaks were observed for participants located in rural settings than those reported for urban settings. This was expected, since in the Mediterranean rural setting, the altitude is often higher and the ambient climate cooler, being consistent with the meteorology of the Mediterranean region. Similar findings were obtained from a study in peri-urban south India^45^. Further, indoor environments (compared to outdoors) were associated with decreased personal air temperatures, being consistent with results from a U.S. population of groundkeepers^46,47^.

However, in the same indoor environments where the median personal air temperatures decreased, corresponding median skin temperatures increased when compared with those skin temperatures obtained outdoors (cross-over trial design). Staying indoors during the study period (summertime) was also associated with a higher number of both personal air-based, and skin-based temperature peaks than those observed outdoors (Fig. S1, Fig. S2), suggesting that participant dynamic occupancy of different indoor (micro)environments was associated with fluctuating air temperatures, influencing people’s susceptibility to indoors overheating^48^. These diurnally varying indoor personal air and skin temperature profiles were observed at random (not under a periodic diurnal pattern) and they were associated with the types of the various behaviors/activities.

Wearable sensor-based skin and personal air temperature values were positively correlated, overall. Recorded median skin temperatures ranged between 33.5 and 36.9 °C, being within the anticipated physiological range of skin temperatures in ambient conditions^49^. Skin temperature gradient may be more responsive to temperature changes in the ambient environment than the body core temperature system. Personal air temperature profiling reflects the variation in the personal air measurements as collected from the breathing zone of the participant within a specific (micro)environment. Skin temperature was higher indoors, whereas ambient personal air temperature was lower when compared to temperatures recorded outdoors for the same participants. This could be explained on the basis of thermoregulation mechanisms sub-optimally operating at a temperature window outside the thermal comfort zone (median indoors personal air temperature of 28 °C). The upper threshold of human thermal comfort for indoor temperatures is typically set at or near 25 °C (95% CI 24.5–26.3 °C)^50^. Among all personal air temperature entries during a 24-h period in this study, the percentage of indoor-based personal air temperature entries higher than 25 °C was 46%, and higher than 26.3 °C was 40%. Sleep time was the most frequent diary entry in both settings (Table S4) with about half of skin peaks associated with sleep (Table S5); indeed, sleep onset was associated with increases in distal and proximal skin temperatures, being characteristic of thermo-setting circadian processes^51,52^. Adults typically spend most of the indoors time at home, including also sleep time^53,54^. The rest 50% of the indoors-located skin temperature peaks were associated with non-sleep activities, such as, being at work or activities inside the house. We observed a 76% decrease in the odds of being indoors, when any activity (surrogate of physical activity) was recorded with the activity tracker, suggesting that the majority of indoor-based behaviours are characterized by a sedentary lifestyle (little or no activity measured with the activity tracker).

It was shown that a diurnally varying temperature profiling was observed indoors for the majority of participants. A higher number of temperature peaks was found in indoor (micro)environments than in outdoors, suggesting higher fluctuations in air temperature levels across different indoor (micro)environments, triggering the detection of such temperature peaks with the sensor logging system. Skin temperature is known to respond quite fast to changes in ambient temperatures (within seconds)^55^. Alternatively, the fact that the majority of participants’ time was spent indoors could be used to explain the higher number of indoors temperature peaks than in outdoors. More than half of adult participants’ time (53%) was spent indoors (in transit and diary-unconfirmed indoors entries were not classified as indoors), but this percentage was lower than those reported in other studies; participants spent about 87% of their time indoors in the USA, based on year-long data^56^, while studies at Birmingham, UK, and Canada reported 72% and 90% of their time being indoors during summer, respectively^24,57^.

Climate change manifestations may be associated with changes in both outdoor and indoor air temperatures (e.g., buildings, houses)^58^. Leaving aside occupational exposures, the evidence on associations between temperatures experienced indoors and health impacts is scarce. There is limited evidence directly linking indoors individual-level temperature measurements with health outcomes and to date, no available data exist on the association between temperature peaks across indoor microenvironments and a health outcome. Literature suggests linkages between indoor air temperatures > 26 °C and increased proportion of respiratory distress calls during summer in the New York city, although this was not a statistically significant trend (*p*** = **0.056)^57^. It was shown that 66% of excess deaths associated with a a large-scale California heat wave in 2006 were at home (RR = 1.12, CI: 1.07–1.16)^59^. For almost 85% of the hot period during the extremely hot summer of 2007 in Greece, indoor temperatures in 50 low-income non-air-conditioned houses exceeded 30 °C, and about 216 continuous hours > 30 °C and six days > 33 °C were recorded in many buildings^60^. During a heat-wave in London, UK, it was reported that 33% of bedrooms reached uncomfortable night-time temperatures of 26 °C or greater^61^. The findings of the pilot non-pharmacological randomized trial (TEMP) showed the positive association between sensor-based skin temperatures and leptin levels in healthy individuals following a short-term stay in climatologically cooler areas during summer^33^. A WHO-led systematic review showed that there was no evidence published after 2003 for indoor temperature monitoring that would allow for a direct link to be established between indoor temperatures and health outcomes, such as all-cause mortality, heatstroke, hyperthermia, dehydration or hospital admission^62^. In effect, current WHO housing and health guidelines regarding maximum indoor temperatures are conditional, due to lack of scientific evidence^63^. In parallel, the most recent WHO-Europe report on heat and health recommends the adoption of interventions to reduce urban overheating^50^.

This *post-hoc* analysis has several strengths. The use of personal temperature sensors allowed for the novel collection of continuous repeated measurements of individual-level temperatures across different indoor (micro)environments. This could help in improving exposure assessment for epidemiological studies monitoring urban areas or areas characterized by extensive spatiotemporal variation in temperature gradient^64^. To the best of our knowledge, this is the first non-pharmacological trial that used such sensors to track personal air and skin temperature gradient fluctuations for peaks identification in indoor and outdoor microenvironments, when participants crossed from one setting to the other (urban vs rural). This study also highlighted the variability in diurnal personal air and skin temperature profiling across different indoor (micro)environments. This indoors-based temperature variance is currently at large dismissed in large epidemiological studies that focus upon the climate and health nexus.

The study has few limitations. The sample size of the study is small, because of the trial’s pilot nature. Moreover, the study period covered only one season (summer), and thus the results cannot be generalized to other seasons. Building/house age, insulation types and materials, ventilation and building space usage have been recognized as important parameters in affecting indoor air temperature variation in different (micro)environments^65^, but such information was not available in this study. Data on average air conditioning use during working hours (68% of participants) and data for usual A/C use during summer by house room type (e.g., kitchen, living room etc.) per setting (89% for urban setting and 27% for rural setting) was available (Table S6). However, information on A/C use on the sampling day and its duration in each setting was lacking. Potential exposure misclassification due to recall bias in diary completion is possible; for example, participants may have noted being outdoors even when less than 30 min was spent outdoors.

The variance in diurnal personal air and skin temperature measurements as recorded with the wearable sensors highlights the inherent complexities of characterizing one’s exposure to various microclimates experienced while spending time in different indoor or outdoor (micro)environments. It is warranted that more accurate temperature monitoring at the individual level via the use of wearable sensors would effectively characterize the temperature-health continuum. Novel methodological frameworks that increasingly find use in environmental health sciences such as that of the human exposome do increasingly utilize individual level sensor data for a multitude of exposures in their study designs^66^. Towards this, exposure modelling approaches, such as the Agent-Based Modelling (ABM) framework, present an opportunity to couple sensor data with the dynamic spatial simulation of different behaviors/activities during one’s busy day in urban areas^67^. Moreover, the behavioral activities recorded from the participants in TEMP trial could help one in better characterizing human activities at the individual level, one of the major challenges faced by the ABM modelling approach.

The practical implications of indoor overheating effects on human health may be high in this global climate crisis era. In the recent 2019 Eurobarometer survey (n = 27,000) in 28 EU Member States, about 93% of EU citizens perceived climate change as a serious problem and 79% perceived it as a very serious problem^68^. The manifestation of indoor overheating accompanied by elevated and variable air temperature gradient profiles in urban dwellings represents an emerging planetary health phenomenon worth of studying in more detail^66^. The planetary health framework attempts to provide further insight on the complex relationship between proximate causes of disease, such as environmental stressors and disease outcomes by taking global and local manifestations of climate change into consideration; the planetary health model and its drivers show that human activities instigate a series of biophysical changes that interact with each other and modify proximate causes of disease, thus, adversely impacting human health^1^. More population studies or personalized medicine approaches focusing on the indoor overheating phenomenon using exposomic tools are warranted, if we were to better characterize the disease phenotype associated with climate change manifestations. A larger sample obtained in other regions and settings would allow for the replication of the observed associations.

## Supplementary Information

Supplementary Information.

Supplementary Data.

## Data Availability

The deidentified datasets of this study can be found in the Supplementary Material. Sensitive data, like postal code were removed prior sharing the current datasets for data protection purposes.

## Abbreviations

IQR: Interquartile range
ASD: Air sensors’ dataset
SSD: Skin sensors’ dataset
ASLD: Air sensors including location dataset
SSLD: Skin sensors including location dataset
PASL: Number of peaks, detected from air sensors’ dataset, including location
PSSL: Number of peaks, detected from skin sensors’ dataset, including location
