# Supplementary material for: A randomized cross-over trial investigating differences in 24-h personal air and skin temperatures using wearable sensors between two climatologically contrasting settings
Source: Sci Rep. 2021 Nov 10;11:22020. doi: 10.1038/s41598-021-01180-y (PMC8580978; doi:10.1038/s41598-021-01180-y)
Supplement: Supplementary file 1 — Supplementary Information. [file 41598_2021_1180_MOESM1_ESM.docx]

**Supplementary Material**

A randomized cross-over trial investigating differences in 24-hour personal air and skin temperatures using wearable sensors between two climatologically contrasting settings

*Andria Constantinou, Stavros Oikonomou, Corina Konstantinou, Konstantinos C. Makris**

^1^ Cyprus International Institute for Environmental and Public Health, Cyprus University of Technology, Limassol, Cyprus

***Corresponding author**: Associate Professor of Environmental Health, Cyprus International Institute for Environmental and Public Health, Cyprus University of Technology, Limassol, Cyprus.

**Telephone**: 357-25002398, **Fax**: 357-25002676

**E-mail**: [konstantinos.makris@cut.ac.cy](mailto:konstantinos.makris@cut.ac.cy)

**Table of Contents**

[1 Supplementary Tables and Figures 3](#_Toc66354986)

[Table S1 3](#_Toc66354987)

[Table S2 3](#_Toc66354988)

[Table S3 5](#_Toc66354989)

[Table S4. 6](#_Toc66354990)

[Table S5 6](#_Toc66354991)

[Table S6 7](#_Toc66354992)

[Fig. S1. 7](#_Toc66354993)

[Fig. S2. 8](#_Toc66354994)

[2 24-hour Recall Activity Diary Analysis 8](#_Toc66354995)

[2.1 Determining what data you can access 8](#_Toc66354996)

[2.2 Obtaining the data 8](#_Toc66354997)

[2.3 Creating the data files 9](#_Toc66354998)

[2.4 Assumptions for extending activities throughout the diary 11](#_Toc66354999)

[3 Diurnal Temperature Peak Profiling: Sensor Data Analysis 15](#_Toc66355000)

[3.1 Determining what data you can access 15](#_Toc66355001)

[3.2 Obtaining the data 15](#_Toc66355002)

[3.3 Cleaning the data 18](#_Toc66355003)

[3.4 Deriving the final inputs 18](#_Toc66355004)

[3.5 Exploratory data analysis 19](#_Toc66355005)

# Supplementary Tables and Figures

Table S1**.** Summary table of activities’ wider groups in each setting and overall.

| **Groups** | **Rural %** | **Urban %** | **Overall %** |
| --- | --- | --- | --- |
| Indoor activities | 45 | 58 | 53 |
| Outdoor activities | 11 | 3 | 6 |
| Unspecified location | 7 | 9 | 9 |
| NA | 37 | 30 | 32 |
| Total | 100% | 100% | 100% |

| Table S2**.** Mixed effect model for the interaction of temperature with setting for personal air and skin sensors. | | | | | | | |
| --- | --- | --- | --- | --- | --- | --- | --- |
|  | **Personal air sensors** | | | **Skin sensors** | | | |
|  | **Location (indoors)** | | | | **Location (indoors)** | | |
| *Predictors* | *Odds Ratios* | *95% CI* | *p* | | *Odds Ratios* | *95% CI* | *p* |
| Temperature | 0.766 | 0.722 – 0.813 | **<0.001** | | 2.616 | 2.471 – 2.769 | **<0.001** |
| settings [Urban] | 3.857 | 3.470 – 4.287 | **<0.001** | | 2.645 | 2.401 – 2.913 | **<0.001** |
| Temperature * setting | 0.652 | 0.589 – 0.721 | **<0.001** | | 0.793 | 0.727 – 0.865 | **<0.001** |
| **Random Effects** | | | | | | | |
| σ^2^ | 3.29 | | | | 3.29 | | |
| τ_00_ | 9.71 _Code_ | | | | 9.79 _Code_ | | |
| ICC | 0.75 | | | | 0.75 | | |
| N | 37 _Code_ | | | | 35 _Code_ | | |
| Observations | 42872 | | | | 39639 | | |
| Marginal R^2^ / Conditional R^2^ | 0.040 / 0.757 | | | | 0.066 / 0.765 | | |

| Table S3**.** Mixed effect models per sensor type (personal air/skin) and setting (urban/rural) | | | | | | | | | | | | | |
| --- | --- | --- | --- | --- | --- | --- | --- | --- | --- | --- | --- | --- | --- |
|  | **Air sensors / urban** | | | **Air sensors / rural** | | | **Skin sensors / urban** | | | **Skin sensors / rural** | | | |
|  | **Location (indoors)** | | | **Location (indoors)** | | | **Location (indoors)** | | | **Location (indoors)** | | | |
| *Predictors* | *Odds Ratio* | *95% CI* | *P* | *Odds Ratio* | *95% CI* | *p* | *Odds Ratio* | *95% CI* | *p* | *Odds Ratio* | *95% CI* | *p* |  |
| Temperature | 0.422 | 0.384 – 0.464 | **<0.001** | 0.518 | 0.482 – 0.556 | **<0.001** | 1.846 | 1.713 – 1.989 | **<0.001** | 3.125 | 2.930 – 3.334 | **<0.001** |  |
| **Random Effects** | | | | | | | | | | | | | |
| σ^2^ | 3.29 | | | 3.29 | | | 3.29 | | | 3.29 | | | |
| τ_00_ | 20.86 _Code_ | | | 2.42 _Code_ | | | 32.18 _Code_ | | | 3.53 _Code_ | | | |
| ICC | 0.86 | | | 0.42 | | | 0.91 | | | 0.52 | | | |
| N | 36 _Code_ | | | 15 _Code_ | | | 32 _Code_ | | | 15 _Code_ | | | |
| Observations | 31527 | | | 11345 | | | 27148 | | | 12491 | | | |
| Marginal R^2^/Conditional R^2^ | 0.024 / 0.867 | | | 0.086 / 0.473 | | | 0.010 / 0.908 | | | 0.169 / 0.599 | | | |

Table S4. Summary table of participant activities (%) in the rural and urban settings during the 24-h time period (based on the reported entries).

|  | **Urban (%)** | **Rural (%)** | |
| --- | --- | --- | --- |
| Sleep | 29.2 | | 31.7 |
| Being at work | 15.9 | | 1.2 |
| Watching TV | 3.5 | | 2.4 |
| Relax | 0.6 | | 1.5 |
| Housekeeping | 1.6 | | 1.9 |
| Being at home^1^ | 4.8 | | 4.5 |
| Outside house^1^ | 1.5 | | 3.3 |
| Walking | 0.4 | | 1.8 |
| Outdoor activities | 0.7 | | 5.7 |
| Breakfast/Lunch/Dinner | 3.5 | | 4 |
| Driving | 4.3 | | 1.6 |
| Other^2^ | 4.35 | | 3.69 |

1: Participants didn’t give the exact activity, but the location. Being at home refers to any activity that took place in the enclosed indoor environment of their house. Outside house refers to any activity that took place outside the enclosed indoor environment of their house.

2: Activities such as reading, cycling, playing indoor and cooking with frequency <1% in the 24-h time period.

Table S5**.** Number and percentage of the peaks (based on skin sensors’ data) detected indoors during sleep and no sleep activities.

|  | **n** | **%** |
| --- | --- | --- |
| Sleep | 70 | 49 |
| No sleep | 73 | 51 |
| Total | 143 | 100 |

Table S6**.** Number and percentages of participants who used on average air conditioning units in any of the following rooms; kitchen, bedroom and living room, in each setting. Also, number and percentages of the participants who used A/C on average during working hours is presented.

| **Average A/C use in either kitchen, bedroom or living room** | **Urban**  **n (%)** | **Rural**  **n (%)** |
| --- | --- | --- |
| Yes | 33 (89) | 10 (27) |
| No | 4 (11) | 27 (73) |
| Average use of A/C during work hours | 25 (68) | - |


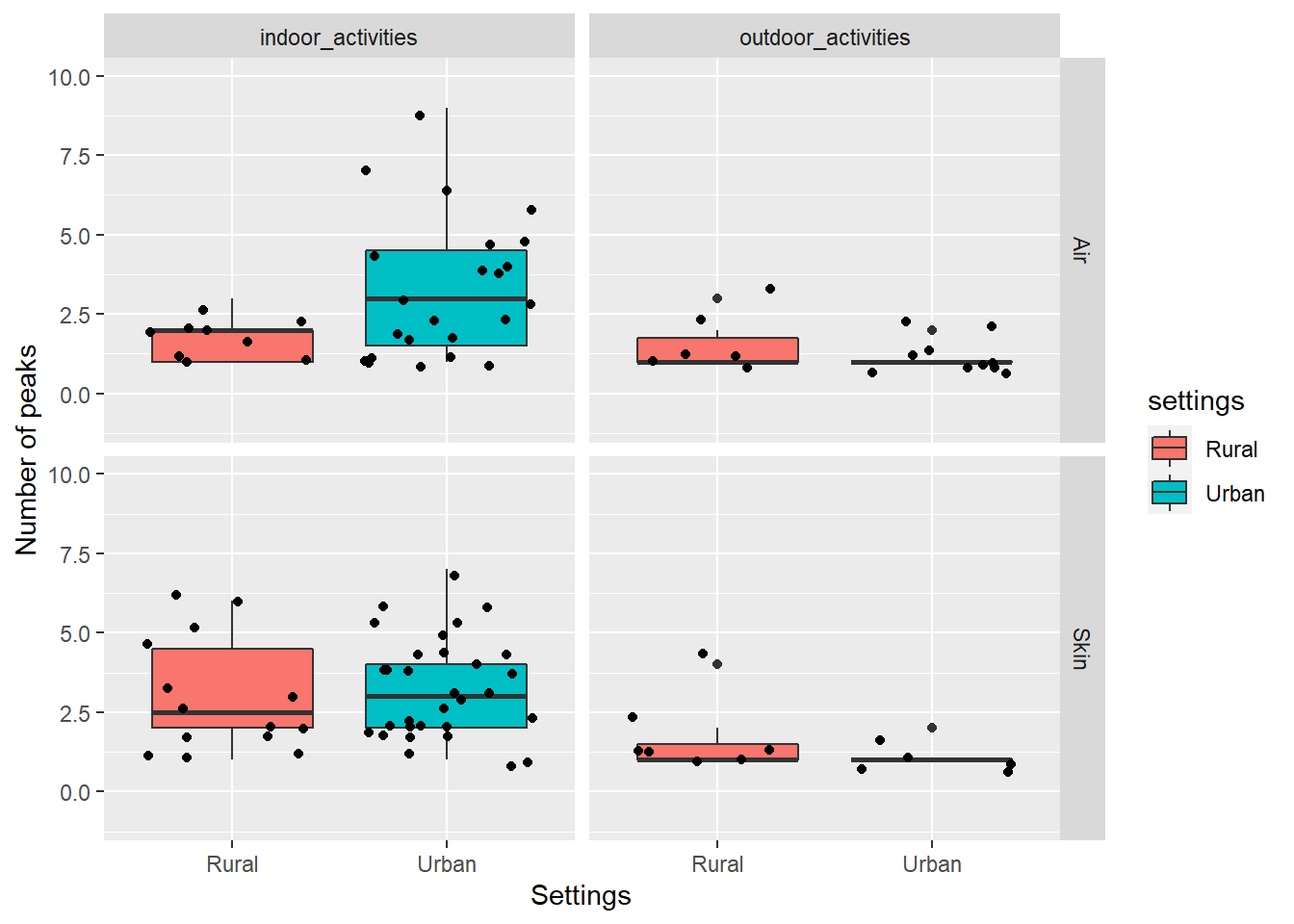


Fig. S1. Number of peaks during indoor and outdoor activities per setting (Rural - Urban) and per sensor type (Personal air - Skin)


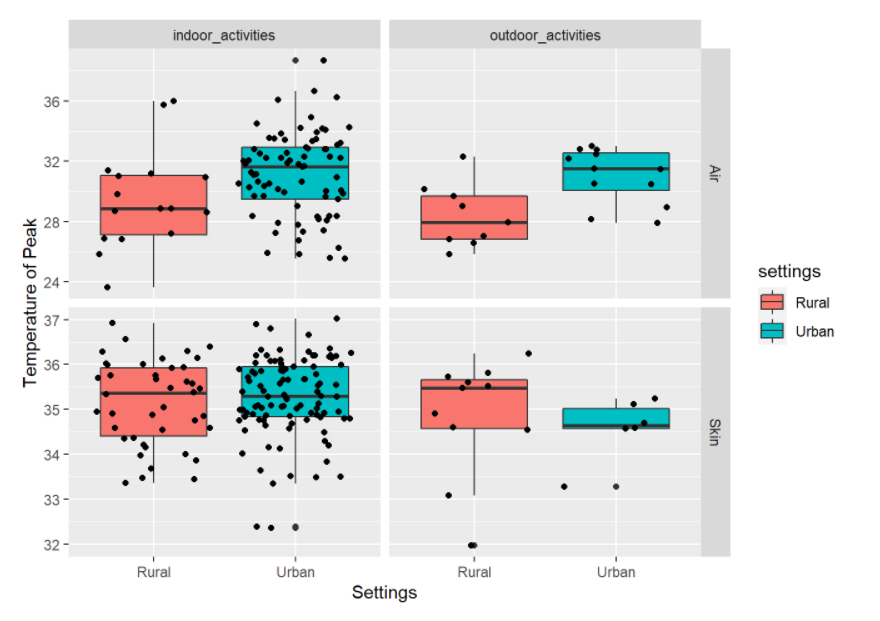


Fig. S2. Raw temperature of the peaks during indoor and outdoor activities per setting (Rural - Urban) and per sensor type (Personal air - Skin)

# 24-hour Recall Activity Diary Analysis

## Determining what data you can access

Existing data contains information about the activities reported from the participants on their sampling day in each of the two settings. Participants recorded their activities in the given timetable with a per 30-minutes period structure.

## Obtaining the data

Data was obtained from 24-hour recall activity diaries which the participants filled out as self-reports in the given diary corresponding to their sampling day in each setting.

## Creating the data files

An Excel file was created in order to digitize the activities as reported in participants’ diaries. In the self-reported activity diaries, the participant had to fill in the type of activity in the corresponding 30-minute interval period. Participants could add the exact starting-ending time of their activity or its duration.

The Excel file reflected the activity diary per minute (1440 entries max), so that data could be merged with the ‘per-minute’ temperatures’ data entries from the personal air/skin sensors. The Excel contained the following variables in order: ID, setting and 1-1440. These variables have the following meaning:

ID: the unique identification number of the study participant

setting: the settings’ order with which the diaries where completed by the participants (1: First urban setting , 2: First rural setting)

1-1440: each number reflects a minute of the sampling’s day starting from 05:00:00

MTUS (Multinational Time Use Study) guidance^1^, was used to deal with diary missing values. Assumptions for filling gaps:

- A gap of 30 or 60 minutes in the activity between two reported activities of different location (e.g. watching TV and then being in the hospital), we assumed that at that period of time, participant was using a mode of transport and marked as ‘unreported transport’.
- A gap of 30 or 60 minutes in the activity between one activity and ‘being at work’ was assumed as a period of time during which the participant was using a mode of transport. The missing data was filled in based on the participant questionnaire responses (which mode of transport do you use for reaching your workplace), where available.
- A missing ‘sleep’ activity during the night and ‘wake up’ activity during morning hours, was filled based on the participant questionnaire responses (what time do you usually go to sleep and what time do you usually wake up in the morning (free or work day for the participant).

The list below explains the changes done in the diary data of those participants who had missing values, based on the above. The deleted diaries for the participants with non-available sensor data, are also listed.

Τ10/1: ‘being at work’ added at 10am and 6pm (working hours 9-5), 20 mins from/to work

Τ10/2: ‘unreported transport’ added between two activities in different locations

Τ11/1: ‘sleep’ added at 23.30 (activity data equals zero) (Sleeping time on free day based on questionnaire data)

Τ11/2: ‘sleep’ added at 23.00 (activity data equals zero) (Sleeping time based on questionnaire data).

Τ12/1: ‘wake up’ added at 6am from questionnaire . (wakes up at 6.20 based on questionnaire data)

T13/1: deleted, no sensor data

T14/1: deleted, no sensor data

Τ14/2: ‘Driving’ added (Enclose vehicle to reach workplace /1h) at 11am and ‘unreported transport’ at 4pm.

T15/2: deleted, no sensor data

Τ16/1: ‘driving’ added at 7.30pm from work to home from questionnaire . Enclose vehicle

T16/2: ‘unreported transport’ added between activities with different location

Τ17/1: ‘unreported transport’ added at 8.30pm. went to festival

T18/1: ‘sleep’ added at 2am from questionnaire.

Τ18/2: ‘sleep’ added at 2am. From questionnaire. Bedtime 2am (free day)

Τ21/2: open vehicle to driving (6.30am) and driving open vehicle at 1.30pm. from questionnaire.

Τ21/1: deleted, no sensor data

Τ22/1: deleted, no sensor data

Τ22/2: ‘unreported transport’ added at 5.30pm, 8pm, 9pm and 12.30am. From/to home cafeteria/restaurant. ‘Sleep’ added at 3am from questionnaire.

T23/1: deleted, no sensor data

Τ29/2: ‘unreported travel’ added at 7pm. And 23.30

Τ29/1: ‘driving’ added at 7am. From questionnaire enclosed vehicle

Τ34/2: deleted, no sensor data (because the sensor stopped measuring)

Τ37/1: ‘unreported transport’ added between activities with different location

Τ37/2: ‘sleep’ added at 24.00 according to questionnaire (free day)

Τ38/2: ‘unreported transport’ added between activities with different location

Τ42/1: ‘sleep’ added at 23.00 according to questionnaire (free day)

Τ42/2: ‘wake up’ added at 8am. According to questionnaire (work day) ‘unreported transport’ added between activities with different location

Τ43/1: ‘unreported transport’ added between activities with different location

T43/2: deleted, no sensor data

Τ45/2: ‘sleep’ added at 24.00 according to questionnaire both free day and work day

Τ46/1: deleted, no sensor data

Τ46/2: ‘wake up’ added at 5.30 according to questionnaire both free day and work day, ‘sleep’ added at 00.00 according to activity data (zero) and questionnaire (free day)

Τ47/1: deleted, no sensor data

Τ47/2: ‘sleep’ added at 00.30am from questionnaire both work day and free day

Τ51/2: ‘wake up’ added at 6am according to questionnaire (work day), ‘being at work’ added until 3pm from questionnaire

Τ51/1: deleted, no sensor data

Τ52/1: deleted, no sensor data

Τ52/2: ‘wake up’ added at 7am according to questionnaire (work day), ‘sleep’ added at 24.00am

T53/2: deleted, no sensor data

Τ54/1: deleted, no sensor data

Τ61/1: deleted, no sensor data

Τ61/2: ‘wake up’ added at 6.30am according to questionnaire (work day)

Τ66/1: deleted, no sensor data

Τ56/1: deleted, no sensor data

Τ64/1: deleted, no sensor data

Τ67/1: deleted, no sensor data

Τ67/2: ‘unreported transport’ added between activities with different location

## Assumptions for extending activities throughout the diary

The file in excel starts from 05:00:00 on the sampling day to 04:59:00 on the sampling’s next day. The time periods were the same as the diary, meaning that there were 48 available time periods per participant to put an entry (one entry per half an hour). Some participants didn’t specify when an activity ended or its duration, so its extension wasn’t possible. Thus, we tried to fill in the empty time periods by making the following assumptions:

- Before the morning ‘wake up’, all cells (each cell corresponds to a time period) were filled with ‘sleep’. After the last entry of night ‘sleep’, all cells were filled with ‘sleep’.
- Empty cells between ‘sleep’ and ‘wake up’, were filled with ‘sleep’.
- Empty cells between ‘Being at work’ and ‘Driving’ (where Driving, based on the diary of each participant refers to the exit from their workplace and car usage) were filled with ‘Being at work’.

The following activities in the sampled 24-h period were recorded and grouped in 3 wide categories:

1. **Indoor activities**

- *Sleep/wake*:

‘Proino xipnima’, ‘xipnisa’ or ‘xipnima’ noted as ‘wake up’ and ‘piga gia ipno’, ‘xaplosa’, ‘kimithika’ or ‘ypnos’ as ‘sleep’.

- *Personal hygiene*:

‘Mpanio’ or ‘ntouz’ will be noted as ‘bath’.

- *Inside house*:

Activities that took place within the surrounding house area.

‘Tileorasi’ or ‘tainia’, ‘douleia ston ipologisti’, ‘doulies tou spitiou’ or ‘katharisma’ or ‘sideroma’ noted as ‘watching TV’, ‘working on the computer’ and ‘housekeeping’, respectively. ‘Eftasa spiti’ or ‘mpika spiti’ or ‘spiti’ or ‘mesa’, ‘Epitrapezia paixnidia sto spiti’ or ‘Drastiriotites me paidia sto spiti’, or ‘drastiriotites’, ‘Chalarosi’, ‘Diavasma’ or ‘Meleti’ and ‘Mageirema’ noted as ‘Being at home’, ‘Playing inside house’, ‘Relax’, ‘Reading’, ‘Cooking’, respectively.

- *Work*:

‘Douleia sto grafeio’, ‘douleia’, ‘ergasiakos xoros’ and ‘ergasia’ noted as ‘being at work’.

- *Indoors to other than home*:

‘Eisodos se ekklisia’, ‘Ekklisia’ ‘Eisodos se magazi’ and ‘Eisodos gia mathima’ noted as ‘Indoor’.

- *Physical activities - indoor:*

‘Gimnastirio’ noted as ‘Gym’.

- *Indoor to other than home – definite use of A/C*:

‘Eisodos se polikatastima’, ‘Soupermarket’, ‘Episkepsi se giatro’, ‘Nosokomio’, ‘Kommotirio’ and ‘Tompola se xenodoxeio’ noted as ‘Indoor department store’, ‘Indoor supermarket’, ‘Indoor doctor’s office’, ‘Indoor hospital’, ‘Indoor hairdressing’ and ‘Playing indoor hotel’, respectively.

1. **Outdoor activities**

- *Outside house*:

Activities that took place outside but within the surrounding house area.

‘vgika exo sti veranta’, ‘anamma karvounon gia souvla’, ‘exo stin avli’, ‘vgika exo’, ‘se exoteriko xoro’, ‘exo ston kipo’ or ‘exo’ or ‘Kipouriki’, noted as ‘Outside house’.

- *Physical activities - outdoor*:

‘Podilasia’, ‘ekana podilato’ ‘peripatos me to podilato’ and ‘perpatima’, ‘vgika na perpatisw’, ‘volta’ or ‘peripatos me ta podia’ noted as ‘cycling’ and ‘walking outside’ respectively.

- *Outdoors other than home*:

‘Exodos apo ekklisia’, ‘Exodos apo ergasia’, ‘Exodos apo doulia’, ‘Exodos apo autokinito’, ‘Plateia xoriou’, ‘Ergasies gia proetimasia festival’ and ‘pisina’ noted as ‘Outdoor activities’.

1. **Unspecified location**

- *Physical activity _ unspecified:*

‘Fisiki askisi’ noted as ‘Physical Exercise’.

- *Food* *intake_unspecified*:

‘Proino’ or any of its synonyms (e.g progevma) noted as ‘breakfast’. ‘Mesimeriano’ or ‘gevma’ noted as ‘lunch’ and ‘bradino’, ‘fagito’ or ‘deipno’ as ‘dinner’.

Also, ‘Kafeteria’ noted as ‘cafeteria’ and ‘Estiatorio’ as ‘Restaurant’. 'Ergasia me to psifidoto' noted as ‘Doing mosaic’.

- *Driving_Unspecified*:

‘Mpika sto autokinito’, ‘autokinito’, ‘odigisa’, ‘odigima’ or ‘sto dromo gia ti douleia’, ‘me autokinito’, ‘eisodos sto autokinito’, ‘sto dromo gia to spiti’ and ‘epistrofi sto spiti me autokinito’ ‘exodos apo doulia or grafeio or ergasia kai eisodos sto autokinito’ noted as ‘driving’.

‘Outdoor’ refers to any activity taking place outside an enclosed indoor environment and ‘Indoor’ referred to any activity taking place inside any enclosed indoor environment. ‘Unspecified location’ referred to those activities where the location is not clearly defined by the participant.

Indoor and Outdoor activities were wider groups that contained all subgroups with activities took place anywhere indoor and outdoor, respectively. For indoor activities, a sub-group created for activities where A/C was on (e.g. hotel, hospital). Activities where the location (indoor or outdoor) was not clearly defined, grouped as ‘Unspecified location’ and were not included in the final analysis.

An expanded version was then created presenting activities per minute from 05:00:00 to 04:59:00, using the built-in rep() function in R.

**Table S7.** A sample of the data sheet which includes the participants’ 24-hour diary entries per minute (expanded).


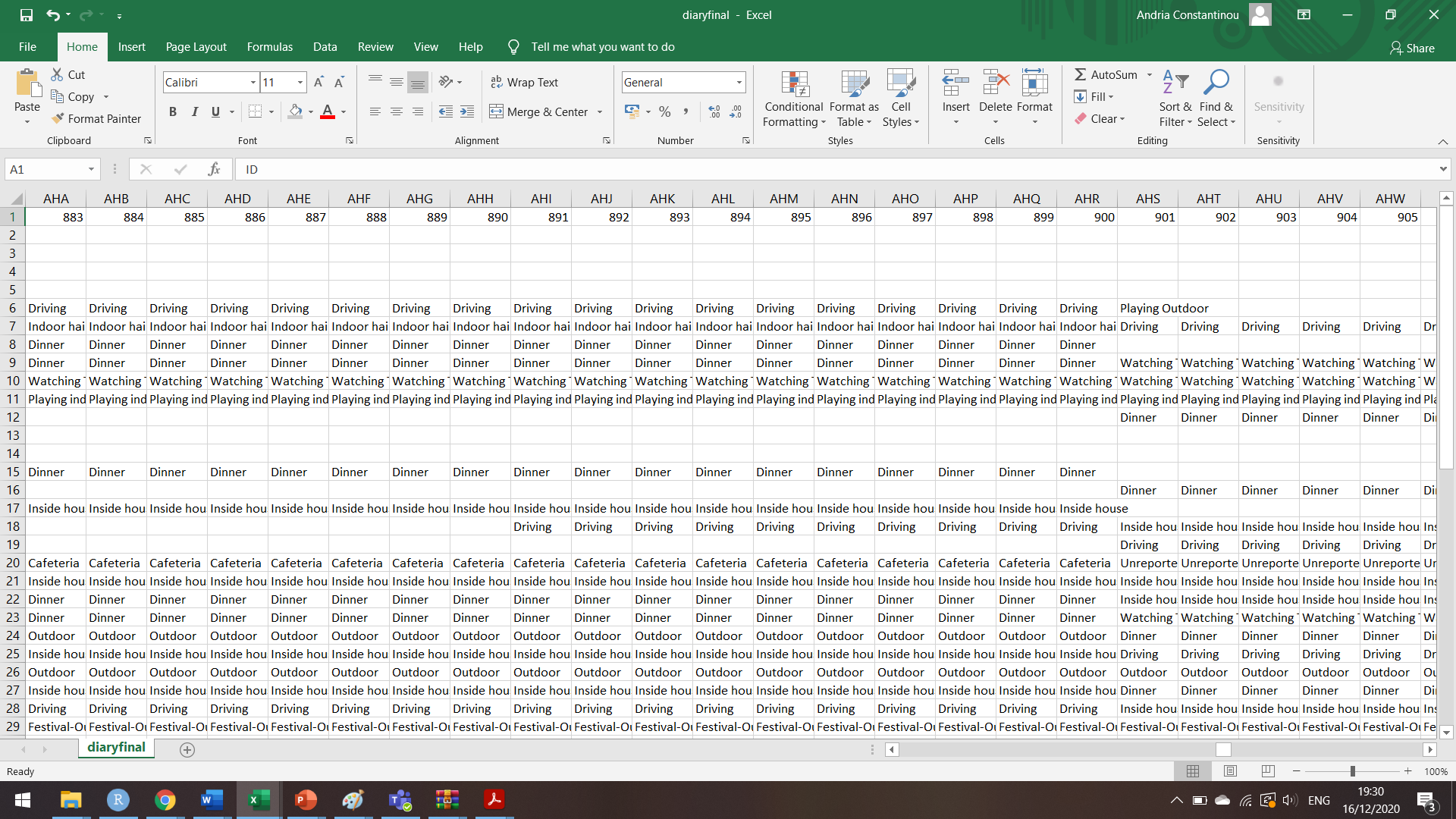

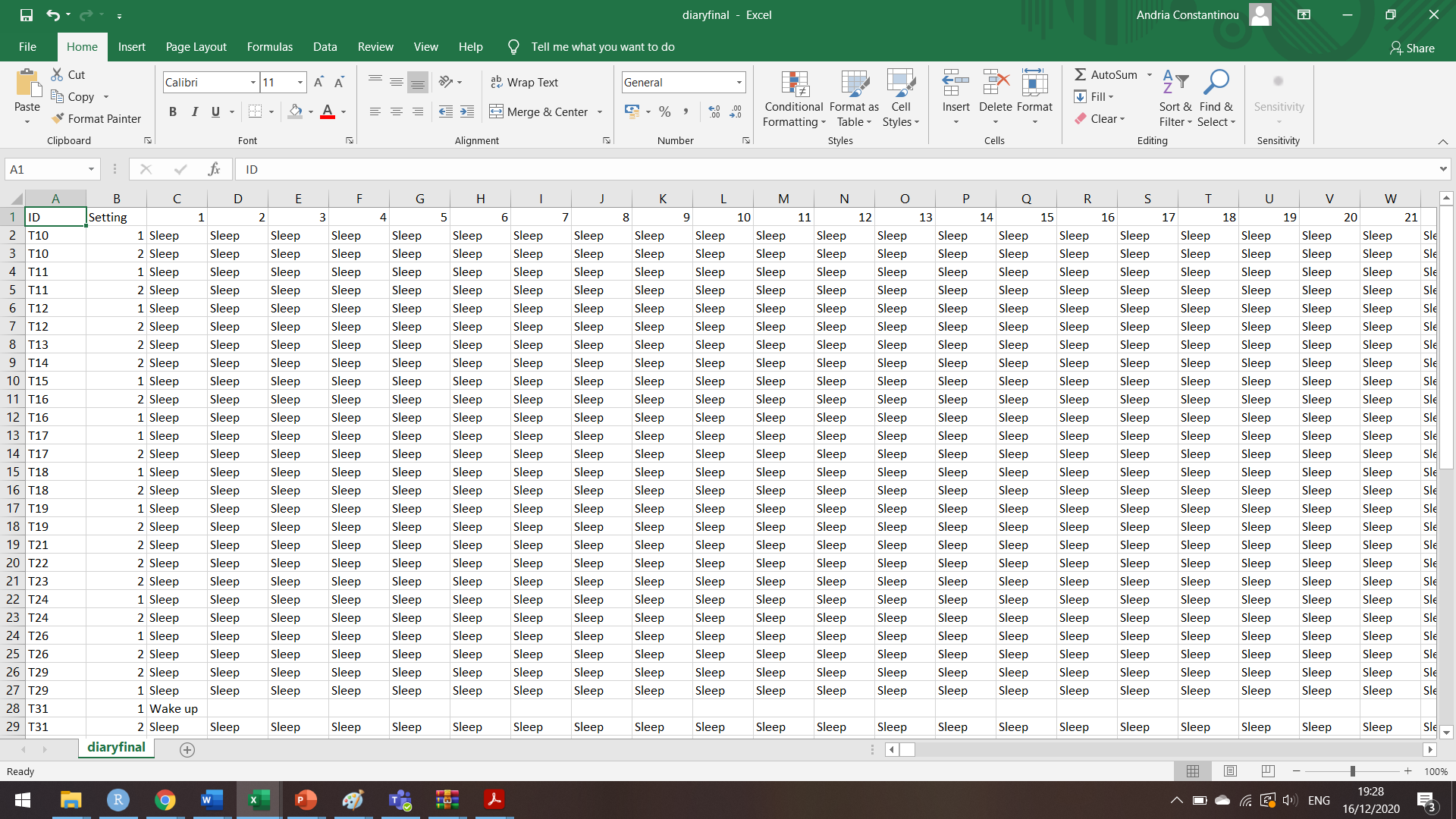

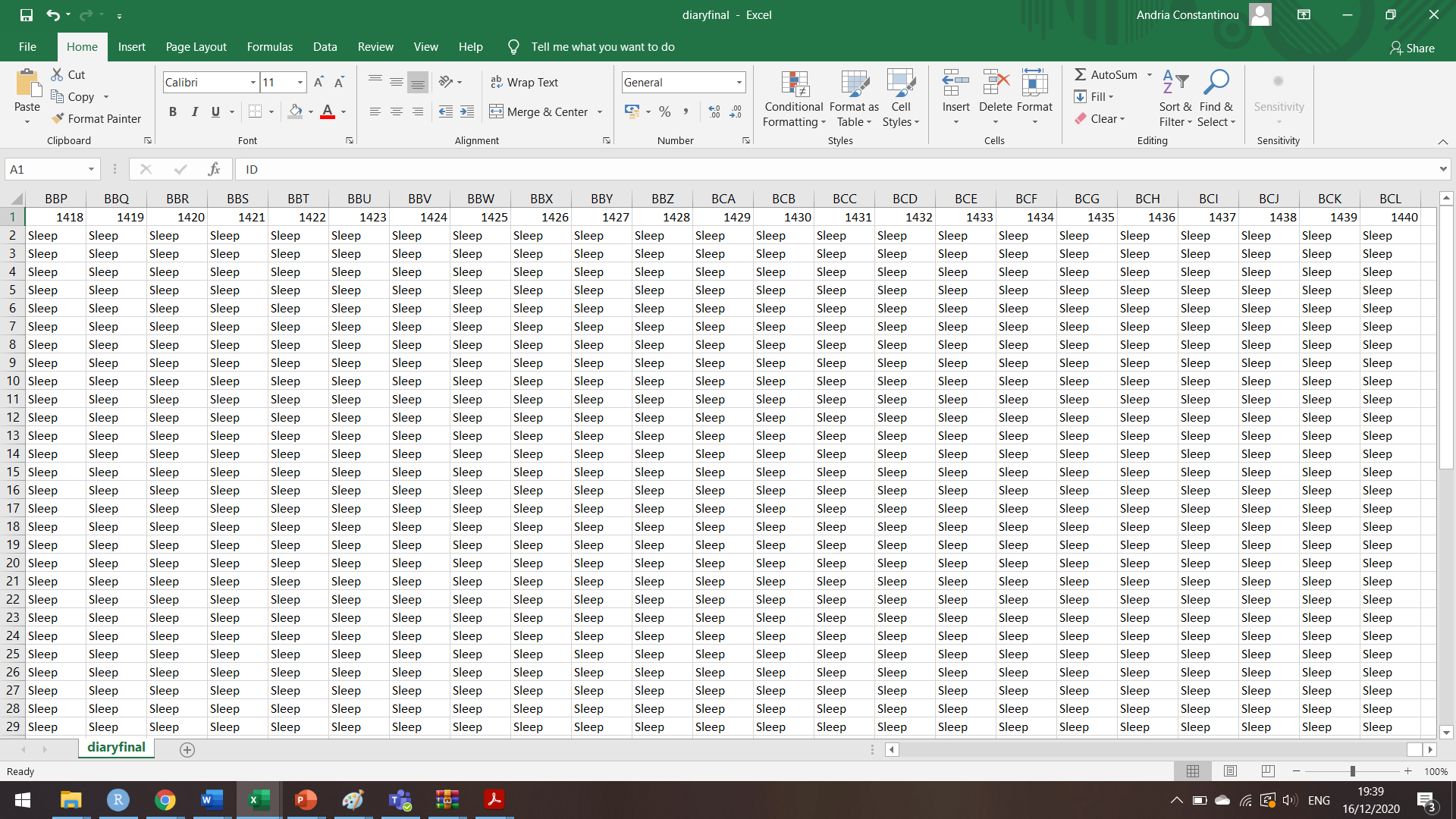


**Table S8.** Summary table of the actual indoor/outdoor activities in the rural and urban settings. In the table the activities’ percentage by location and setting (based on the reported entries) is presented.

| **Indoor activities** | Rural% | Urban% |  | **Outdoor activities** | Rural% | Urban% |
| --- | --- | --- | --- | --- | --- | --- |
| Sleep | 71 | 50 |  | Outside house^1^ | 30 | 50 |
| Being at work | 3 | 27 |  | Walking | 16 | 12 |
| Watching TV | 5 | 6 |  | Cycling | - | 10 |
| Relax | 3 | 1 |  | Outdoor activities | 50 | 24 |
| Housekeeping | 4 | 3 |  | Driving open vehicle | - | 4 |
| Being at home^1^ | 10 | 8 |  | Lunch | 2 | - |
| Other^2^ | 4 | 5 |  | Dinner | 2 | - |

1: Participants didn’t give the exact activity but the location. Being at home refers to any activity that took place in the enclosed indoor environment of their house. Outside house refers to any activity that took place outside the enclosed indoor environment of their house.

2: Activities such as reading, playing and cooking with frequency <2%.

# Diurnal Temperature Peak Profiling: Sensor Data Analysis

## Determining what data you can access

Personal air and skin temperature data obtained with the personal eTact© sensors were smoothed by 10% (smoothing span=0.1) using local polynomial regression (LOESS) analysis^2^, to have the most possible realistic output^3^. Smoothed data were used to generate the temperature peak profiling. 24-hour data was generated in each sampling day of each of the two settings (one in urban and the other in the mountainous setting) for both skin and air temperature.

## Obtaining the data

Data exported from the sensors and imported into R (R (v. 3.6.3) and R studio (v. 1.2.5033)).

In order to obtain the temperature peaks for the skin and air sensors, a peak finding named findpeaks() algorithm was used:

findpeaks (x, nups = 1, ndowns = 0, minpeakheight = 0, minpeakdistance = 1, threshold, sortstr = FALSE)

The parameters have the following meaning:

- ​​x: dataset
- nups (minimum number of increasing steps before a peak is reached) = 1
- ndowns (minimum number of decreasing steps after the peak) = 0
- minpeakdistance (the minimum distance (in our case minutes) peaks have to have to be counted threshold the minimum) = 1
- threshold (the minimum) ​
- sortstr (logical; should the peaks be returned sorted in decreasing order of their maximum value) = FALSE

The threshold takes values from 0.1 to 1. In order to find the optimum threshold value, a sensitivity analysis was done in priori. All participants’ datasets, from both settings, were used for the identification of the number of peaks, using findpeaks() function and changing threshold by 0.1 each time (from 0.1 to 0.7). The median number of peaks derived from each participant, using the different thresholds, was calculated and then a graph was prepared, for each sensor type, to decide which was the optimum value to be used in the final analysis.


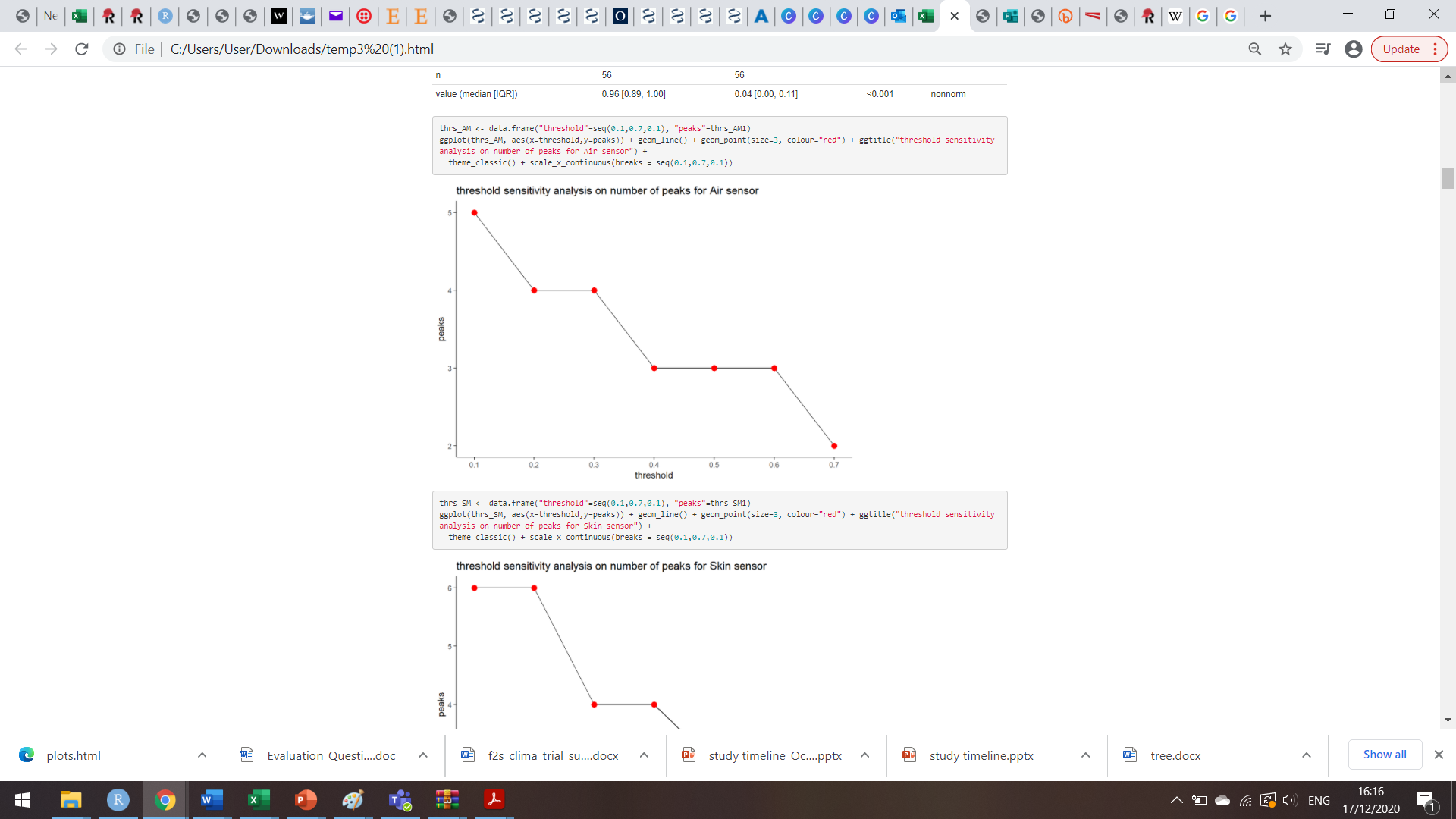


**Figure S3.** Median number of peaks, from all participants’ air sensors datasets, using different thresholds (0.1-0.7)


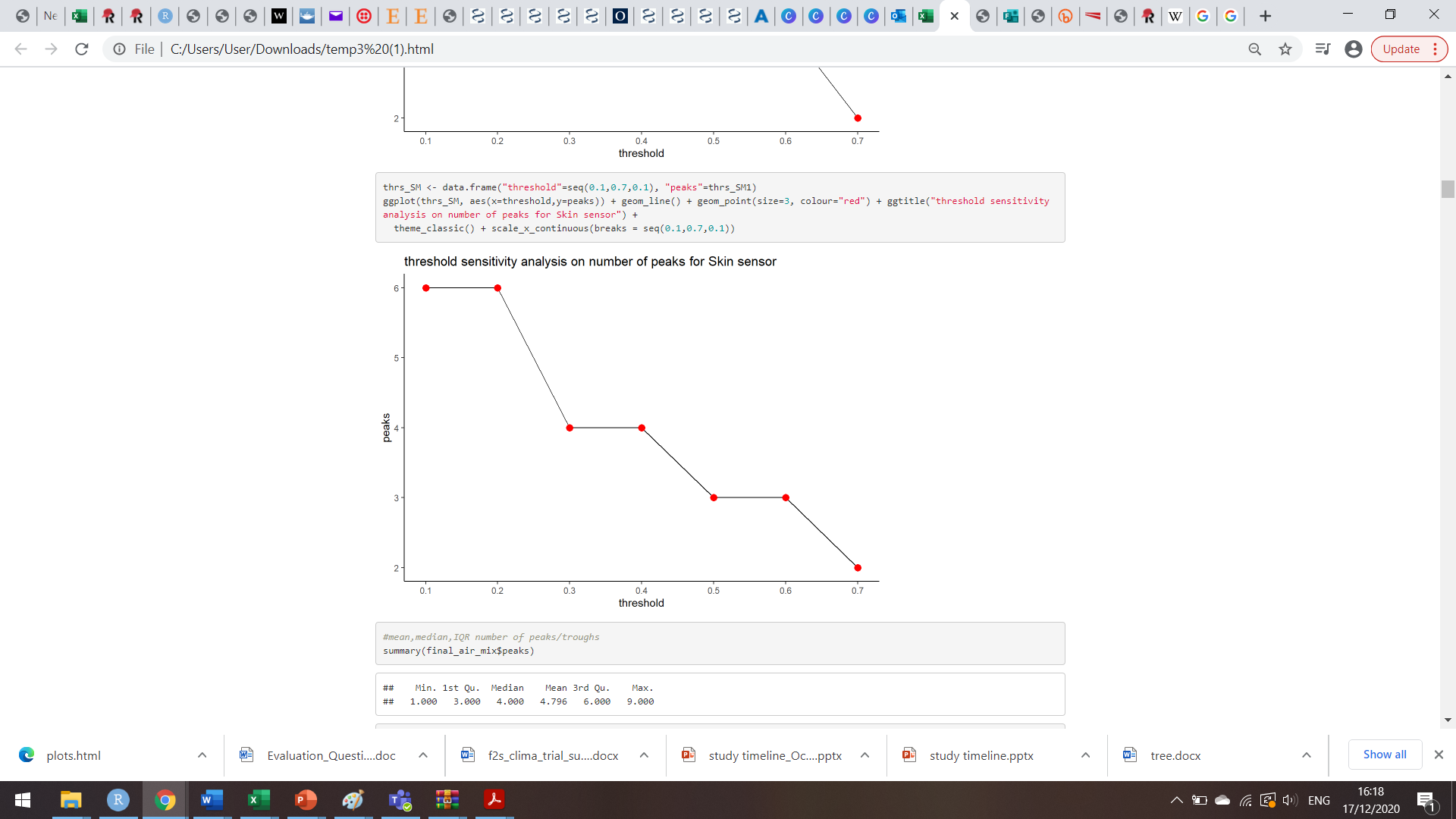


**Figure S4.** Median number of peaks, from all participants’ skin sensors datasets, using different thresholds (0.1-0.7)

Findpeaks() algorithm run using a threshold=0.2 (same for both sensors’ types) and then the number of peaks and their median were identified for both sensors’ types per setting. The threshold 0.2 was the optimum for our analysis since it’s not very sensitive and thus, it didn’t give extreme numbers of peaks but in the same time provides the most realistic output by identifying satisfied number of peaks.

**Figure S5.** Number of peaks per setting (Rural - Urban) and per sensor type (Personal air - Skin).

For each participant, all temperature peak values and the timestamp of it (a number from 1 to 1440 that reflects the time from 05:00:00 of the sampling’s day to 04:59:00 of sampling’s next day) were recorded for skin and personal air temperature.

## Cleaning the data

Of the 53 days’ long duration of sensor data collection for all 39 participants, 5 of the days’ recordings were not optimal. In all of the participants, the last data entry (which
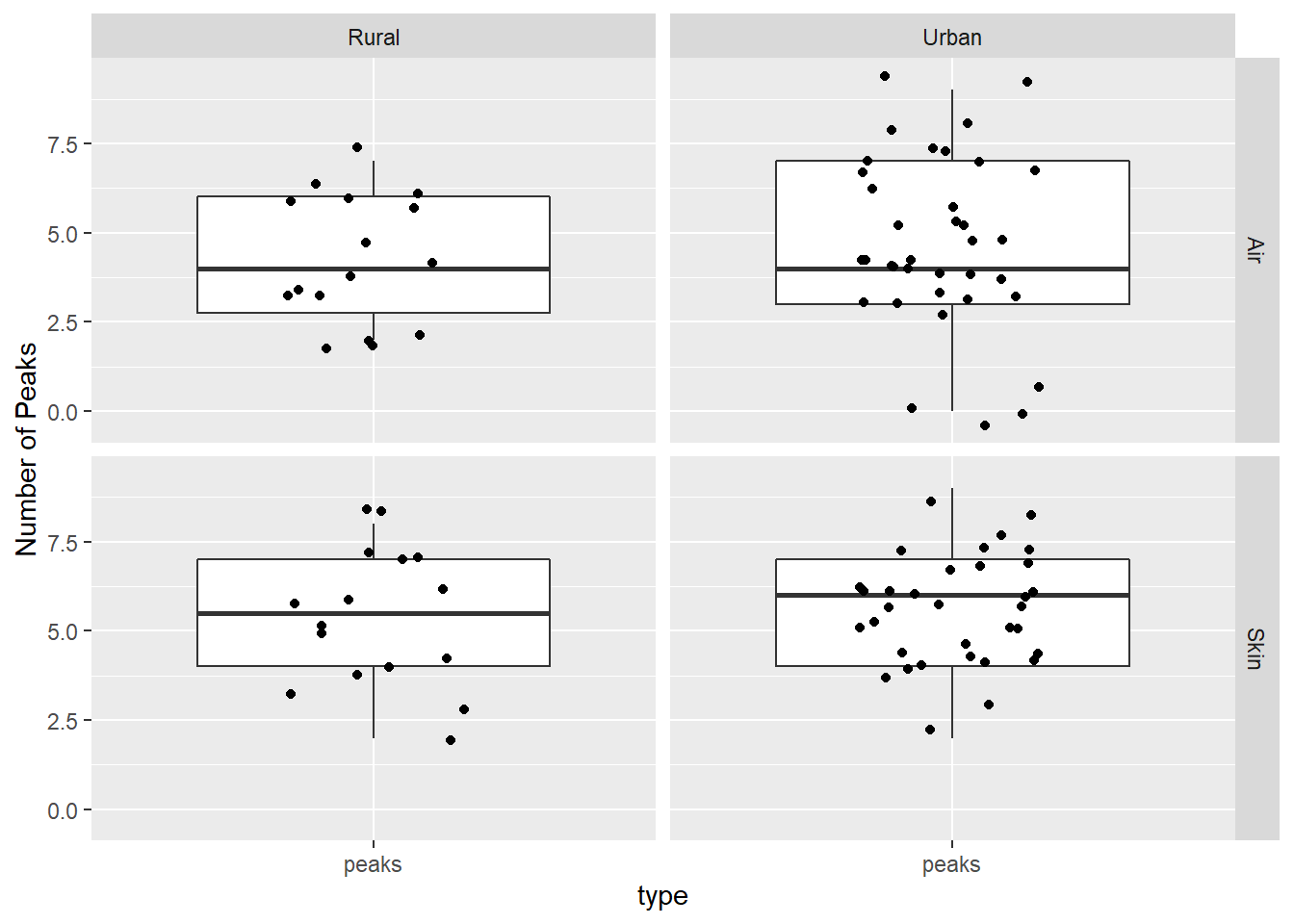
reflected a minute’s worth of time) was deleted since it reflected the time point that the day ended. Thus, all time data spanned from 05:00:00 to 04:59:00 instead of 05:00:00 to 05:00:00.

## Deriving the final inputs

From the given output (after using findpeaks() function), the timestamp at which the peak occurred and the temperature value of the peak were included in the PASL.

## Exploratory data analysis

Temperature peaks were identified across the 24-hour period using the findpeaks() function, with threshold = 0.2 (the other parameters used as default).


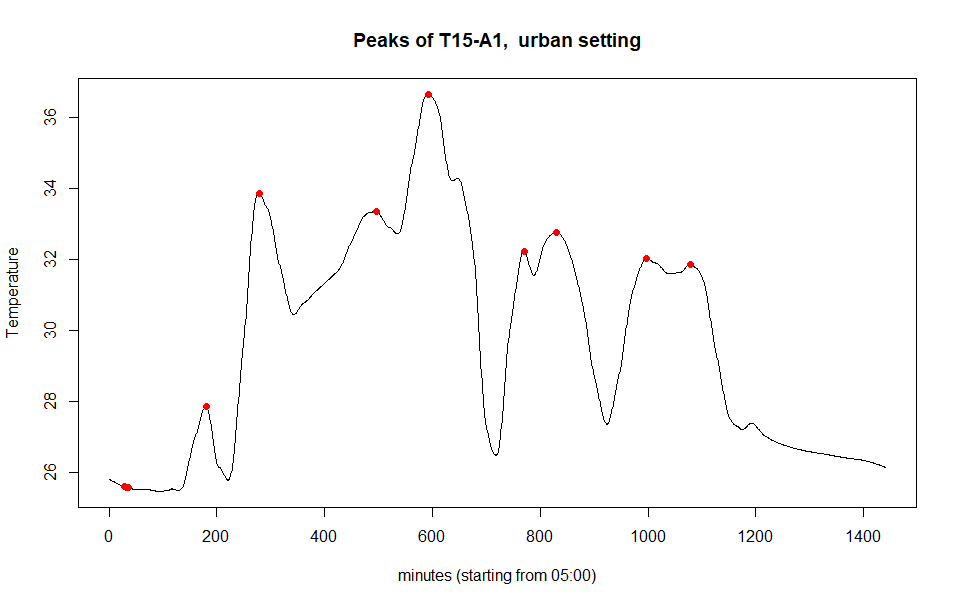

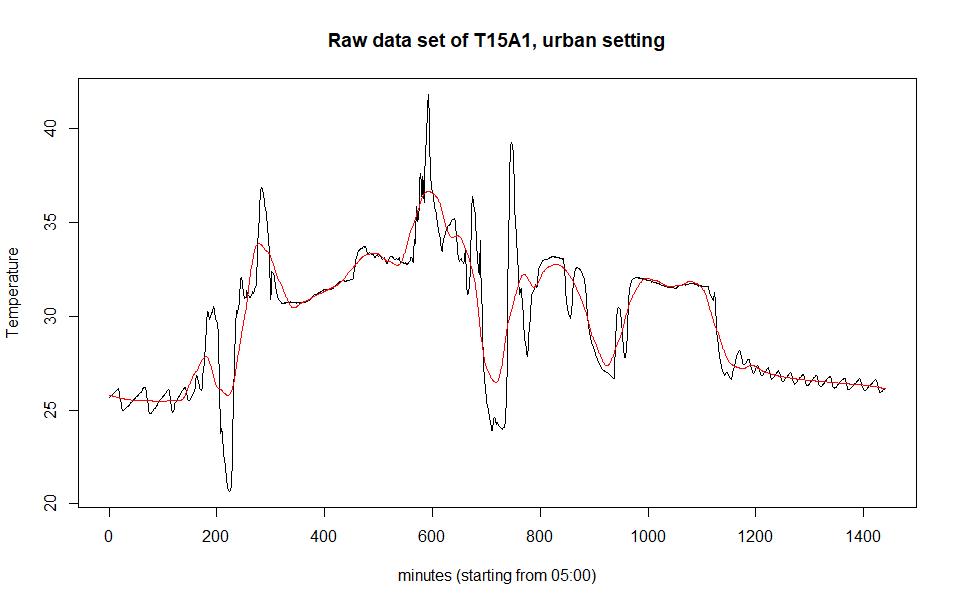


A

B

**Figure S6.** A: Sample graph of personal air temperature sensor data across a 24-hour period in urban setting for a participant (T15). The red line represents the smoothed temperature data. B: The smoothed temperature data of the same participant (black line) and red circles indicate the instances that the algorithm identified as a peak.

**References**

1. Fisher, K. *et al.* Multinational Time Use Study. **4**, (2012).

2. Cleveland, W. S. & Devlin, S. J. Locally Weighted Regression : An Approach to Regression Analysis by Local Fitting Locally Weighted Regression : An Approach to Regression Analysis by Local Fiiting. 37–41 (1988).

3. Prabhakaran, S. r-statistics.co. http://r-statistics.co/Loess-Regression-With-R.html (2016).
