# Supplementary material for: A randomized cross-over trial investigating differences in 24-h personal air and skin temperatures using wearable sensors between two climatologically contrasting settings
Source: Sci Rep. 2021 Nov 10;11:22020. doi: 10.1038/s41598-021-01180-y (PMC8580978; doi:10.1038/s41598-021-01180-y)
Supplement: Supplementary file 2 — Supplementary Data. [file 41598_2021_1180_MOESM2_ESM.zip › New folder/tempOR.html]

Temp study


# Temp study

#### Stavros Oikonomou

#### March 2021

### Table 1 - Baseline characteristics - Study participants (overall and by group)

Baseline characteristics of participants

|  | Overall |
| --- | --- |
| n | 37 |
| Age (mean (SD)) | 41.58 (10.45) |
| Sex = Male (%) | 14 (37.8) |
| BMI (mean (SD)) | 24.84 (3.62) |
| BMIcat (%) |  |
| underweight | 1 ( 2.7) |
| normal weight | 21 (56.8) |
| overweight | 12 (32.4) |
| obese | 3 ( 8.1) |
| education\_level (%) |  |
| Secondary | 10 (27.0) |
| University/college | 13 (35.1) |
| Master/PhD | 14 (37.8) |
| Chronotypecat (%) |  |
| early | 6 (23.1) |
| intermediate | 18 (69.2) |
| late | 2 ( 7.7) |
| Smoking\_status (%) |  |
| Smoker | 7 (18.9) |
| Non-smoker | 25 (67.6) |
| Former smoker | 5 (13.5) |
| alcohol\_freq (%) |  |
| Weekly | 16 (43.2) |
| Monthly | 7 (18.9) |
| Rarely/Never | 14 (37.8) |
| Physical\_exercise = No (%) | 19 (51.4) |
| screen\_hours\_day (mean (SD)) | 5.93 (3.76) |
| days\_mountain (mean (SD)) | 7.05 (2.49) |
| washout\_days (mean (SD)) | 14.92 (9.05) |

Baseline characteristics of participants by group

|  | First urban | First mountainous | p | test |
| --- | --- | --- | --- | --- |
| n | 11 | 26 |  |  |
| Age (mean (SD)) | 39.80 (6.83) | 42.27 (11.58) | 0.533 |  |
| Sex = Male (%) | 2 (18.2) | 12 (46.2) | 0.150 | exact |
| BMI (mean (SD)) | 25.06 (4.46) | 24.75 (3.29) | 0.818 |  |
| BMIcat (%) |  |  | 0.459 | exact |
| underweight | 1 ( 9.1) | 0 ( 0.0) |  |  |
| normal weight | 5 (45.5) | 16 (61.5) |  |  |
| overweight | 4 (36.4) | 8 (30.8) |  |  |
| obese | 1 ( 9.1) | 2 ( 7.7) |  |  |
| education\_level (%) |  |  | 0.189 | exact |
| Secondary | 3 (27.3) | 7 (26.9) |  |  |
| University/college | 6 (54.5) | 7 (26.9) |  |  |
| Master/PhD | 2 (18.2) | 12 (46.2) |  |  |
| Chronotypecat (%) |  |  | 0.009 | exact |
| early | 5 (50.0) | 1 ( 6.2) |  |  |
| intermediate | 4 (40.0) | 14 (87.5) |  |  |
| late | 1 (10.0) | 1 ( 6.2) |  |  |
| Smoking\_status (%) |  |  | 0.867 | exact |
| Smoker | 2 (18.2) | 5 (19.2) |  |  |
| Non-smoker | 7 (63.6) | 18 (69.2) |  |  |
| Former smoker | 2 (18.2) | 3 (11.5) |  |  |
| alcohol\_freq (%) |  |  | 0.893 | exact |
| Weekly | 4 (36.4) | 12 (46.2) |  |  |
| Monthly | 2 (18.2) | 5 (19.2) |  |  |
| Rarely/Never | 5 (45.5) | 9 (34.6) |  |  |
| Physical\_exercise = No (%) | 8 (72.7) | 11 (42.3) | 0.151 | exact |
| screen\_hours\_day (mean (SD)) | 6.82 (3.49) | 5.56 (3.87) | 0.358 |  |
| days\_mountain (mean (SD)) | 6.73 (1.10) | 7.19 (2.90) | 0.611 |  |
| washout\_days (mean (SD)) | 14.55 (9.28) | 15.08 (9.13) | 0.873 |  |

### Summary table for ambient air temperature by setting taking in account all observations

Summary table of ambient air temperature by setting


|  | 1 | 2 | p | test |
| --- | --- | --- | --- | --- |
| n | 51610 | 23040 |  |  |
| Temperature (median [IQR]) | 28.62 [27.14, 30.51] | 27.05 [25.36, 29.19] | <0.001 | nonnorm |

### Summary table for skin temperature by setting taking in account all observations

Summary table of skin temperature by setting


|  | 1 | 2 | p | test |
| --- | --- | --- | --- | --- |
| n | 44967 | 20301 |  |  |
| Temperature (median [IQR]) | 34.64 [33.88, 35.39] | 34.61 [33.82, 35.34] | <0.001 | nonnorm |

### Summary table of cumulative activity by settting based on skin sensors

Summary table of cumulative activity per day based on skin sensors

|  | 1 | 2 | p | test |
| --- | --- | --- | --- | --- |
| n | 44967 | 20301 |  |  |
| Activity (median [IQR]) | 1.00 [0.00, 67.00] | 0.00 [0.00, 76.00] | 0.001 | nonnorm |

```
ggplot(data_air, aes(x=Code_order, y=Temperature, fill=settings)) + geom_boxplot() + ggtitle("temperature boxplot for all code orders on skin data")
```

```
ggplot(data_skin, aes(x=Code_order, y=Temperature, fill=settings)) + geom_boxplot() + ggtitle("temperature boxplot for all code orders on skin data")
```

### Summary table of proportion of Indoor/Outdoor

Summary table of proportion of Indoor/Outdoor

|  | indoor | outdoor | p | test |
| --- | --- | --- | --- | --- |
| n | 56 | 56 |  |  |
| value (median [IQR]) | 95.46 [87.66, 100.00] | 4.54 [0.00, 12.35] | <0.001 | nonnorm |

```
#air sensor peaks both settings
qqnorm(peaks_air_all$P_temperature)
```

```
hist(peaks_air_all$P_temperature)
```

```
#skin sensor peaks both settings
qqnorm(peaks_skin_all$P_temperature)
```

```
hist(peaks_skin_all$P_temperature)
```

```
# qqplot for raw data temperature
ggqqplot(data_air$Temperature)
```

```
ggqqplot(data_skin$Temperature)
```

```
# number of peaks
qqnorm(final_air_mix$peaks)
```

```
hist(final_air_mix$peaks)
```

```
hist(final_skin_mix$peaks)
```

```
qqnorm(data_skin$Activity)
```

```
qqnorm(log(data_skin$Activity + 1))
```

### Boxplot peaks

# Temperature plots for Location Settings

Mean Temperature values of peaks for Air Sensors Urban

| Location | Mean\_Temperature |
| --- | --- |
| indoor\_activities | 31.08767 |
| outdoor\_activities | 31.00878 |
| unspecified\_location | 30.78904 |
| NA | 30.40207 |

Mean Temperature values of peaks for Air Sensors Rural

| Location | Mean\_Temperature |
| --- | --- |
| indoor\_activities | 29.47073 |
| outdoor\_activities | 28.24380 |
| unspecified\_location | 32.41281 |
| NA | 28.68506 |

### Skin Sensor

Mean Temperature values of peaks for Skin Sensors Urban

| Location | Mean\_Temperature |
| --- | --- |
| indoor\_activities | 35.26257 |
| outdoor\_activities | 34.58493 |
| unspecified\_location | 35.23038 |
| NA | 35.03551 |

Mean Temperature values of peaks for Skin Sensors Rural

| Location | Mean\_Temperature |
| --- | --- |
| indoor\_activities | 35.13706 |
| outdoor\_activities | 34.88765 |
| unspecified\_location | 35.57367 |
| NA | 35.17114 |

### Summary table of peak temperature per setting on air sensors

Summary table of peak temperature by setting for air sensors


|  | Rural | Urban | p | test |
| --- | --- | --- | --- | --- |
| n | 26 | 92 |  |  |
| P\_temperature (median [IQR]) | 28.76 [26.90, 30.72] | 31.34 [29.59, 32.79] | 0.001 | nonnorm |

### Summary table of peak temperature per setting on skin sensors

Summary table of peak temperature by setting for skin sensors


|  | Rural | Urban | p | test |
| --- | --- | --- | --- | --- |
| n | 54 | 109 |  |  |
| P\_temperature (median [IQR]) | 35.36 [34.54, 35.80] | 35.25 [34.79, 35.89] | 0.430 | nonnorm |

### Summary table of number of peaks at Indoor/Outdoor location per setting on air sensors

Summary table of number of peaks by setting for air sensor at Indoor/Outdoor location


|  | Rural | Urban | p | test |
| --- | --- | --- | --- | --- |
| n | 17 | 35 |  |  |
| num\_of\_peaks (median [IQR]) | 1.00 [1.00, 2.00] | 2.00 [1.00, 4.00] | 0.113 | nonnorm |

### Summary table of number of peaks at Indoor/Outdoor location per setting on skin sensors

Summary table of number of peaks by setting for skin sensor at Indoor/Outdoor location


|  | Rural | Urban | p | test |
| --- | --- | --- | --- | --- |
| n | 21 | 36 |  |  |
| num\_of\_peaks (median [IQR]) | 2.00 [1.00, 4.00] | 3.00 [2.00, 4.00] | 0.236 | nonnorm |

### Temperature and Activity correlation

```
# ploting Temperature and Activity.
plot(TNUMSKIN_l$Temperature, TNUMSKIN_l$Activity)
```

```
#correlation between Temperature and Activity for Urban
cor.test(TNUMSKIN_l[TNUMSKIN_l$settings=="1","Temperature"],TNUMSKIN_l[TNUMSKIN_l$settings=="1","Activity"], method="spearman", exact=F)
```

```
## 
##  Spearman's rank correlation rho
## 
## data:  TNUMSKIN_l[TNUMSKIN_l$settings == "1", "Temperature"] and TNUMSKIN_l[TNUMSKIN_l$settings == "1", "Activity"]
## S = 4.1904e+12, p-value < 2.2e-16
## alternative hypothesis: true rho is not equal to 0
## sample estimates:
##       rho 
## -0.256576
```

```
#correlation between Temperature and Activity for Rural
cor.test(TNUMSKIN_l[TNUMSKIN_l$settings=="2","Temperature"],TNUMSKIN_l[TNUMSKIN_l$settings=="2","Activity"], method="spearman", exact=F)
```

```
## 
##  Spearman's rank correlation rho
## 
## data:  TNUMSKIN_l[TNUMSKIN_l$settings == "2", "Temperature"] and TNUMSKIN_l[TNUMSKIN_l$settings == "2", "Activity"]
## S = 4.2869e+11, p-value < 2.2e-16
## alternative hypothesis: true rho is not equal to 0
## sample estimates:
##        rho 
## -0.3197906
```

### Summary table for ambient air temperature by setting taking in account all observations

Summary table of ambient air temperature by setting


|  | outdoor\_activities | indoor\_activities | p | test |
| --- | --- | --- | --- | --- |
| n | 3176 | 39696 |  |  |
| Temperature (median [IQR]) | 28.52 [26.79, 30.65] | 27.95 [26.37, 30.27] | <0.001 | nonnorm |

### Summary table for skin temperature by setting taking in account all observations

Summary table of skin temperature by setting


|  | outdoor\_activities | indoor\_activities | p | test |
| --- | --- | --- | --- | --- |
| n | 3986 | 35653 |  |  |
| Temperature (median [IQR]) | 33.92 [32.87, 34.77] | 34.81 [34.04, 35.57] | <0.001 | nonnorm |

### Summary table of cumulative activity by settting based on skin sensors

Summary table of cumulative activity per day based on skin sensors


|  | outdoor\_activities | indoor\_activities | p | test |
| --- | --- | --- | --- | --- |
| n | 3986 | 35653 |  |  |
| Activity (median [IQR]) | 70.50 [1.00, 267.00] | 0.00 [0.00, 7.00] | <0.001 | nonnorm |

### Summary table of peak temperature per setting on air sensors

Summary table of peak temperature by setting for air sensors


|  | indoor\_activities | outdoor\_activities | p | test |
| --- | --- | --- | --- | --- |
| n | 97 | 21 |  |  |
| P\_temperature (median [IQR]) | 30.98 [28.67, 32.78] | 30.13 [27.94, 32.17] | 0.167 | nonnorm |

### Summary table of peak temperature per setting on skin sensors

Summary table of peak temperature by setting for skin sensors


|  | indoor\_activities | outdoor\_activities | p | test |
| --- | --- | --- | --- | --- |
| n | 146 | 17 |  |  |
| P\_temperature (median [IQR]) | 35.29 [34.76, 35.95] | 34.91 [34.59, 35.52] | 0.104 | nonnorm |

### Summary table of number of peaks at Indoor/Outdoor location per setting on air sensors

Summary table of number of peaks by setting for air sensor at Indoor/Outdoor location


|  | outdoor\_activities | indoor\_activities | p | test |
| --- | --- | --- | --- | --- |
| n | 17 | 35 |  |  |
| num\_of\_peaks (median [IQR]) | 1.00 [1.00, 1.00] | 2.00 [1.00, 4.00] | 0.001 | nonnorm |

### Summary table of number of peaks at Indoor/Outdoor location per setting on skin sensors

Summary table of number of peaks by setting for skin sensor at Indoor/Outdoor location


|  | outdoor\_activities | indoor\_activities | p | test |
| --- | --- | --- | --- | --- |
| n | 12 | 45 |  |  |
| num\_of\_peaks (median [IQR]) | 1.00 [1.00, 1.25] | 3.00 [2.00, 4.00] | <0.001 | nonnorm |

### MODELS

#### Reference for Location is “outdoor\_activities” and “Rural” for setting for all 4 dataframes

```
model_1a <-  glmer(Location ~ settings + logTemperature + (1|Code),data= TNUMAIR_l, family = binomial(link="logit")) 

model_1b <- glmer(Location ~ settings + logTemperature + Activity_Group + (1|Code), data= TNUMSKIN_l, family = binomial(link="logit"))

tab_model(model_1a, model_1b, digits=3, title="Mixed Effect Models for Location~ Settings + Temperature + Activity(for skin sensor) + random(Participant) for raw data air and skin sensor")
```

Mixed Effect Models for Location~ Settings + Temperature + Activity(for skin sensor) + random(Participant) for raw data air and skin sensor

|  | Location | | | Location | | |
| Predictors | Odds Ratios | CI | p | Odds Ratios | CI | p |
| (Intercept) | 51.359 | 17.971 – 146.780 | **<0.001** | 165.482 | 54.432 – 503.091 | **<0.001** |
| settings [Urban] | 3.541 | 3.200 – 3.918 | **<0.001** | 3.079 | 2.795 – 3.392 | **<0.001** |
| logTemperature | 0.664 | 0.632 – 0.699 | **<0.001** | 2.065 | 1.972 – 2.162 | **<0.001** |
| SD (Intercept) | 21.093 |  |  | 21.953 |  |  |
| SD (Observations) | 2.718 |  |  | 2.718 |  |  |
| Activity\_Group [Any\_Activity] |  |  |  | 0.211 | 0.193 – 0.230 | **<0.001** |
| Random Effects | | | | | | |
| σ2 | 3.29 | | | 3.29 | | || τ00 | 9.30 Code | | | 9.54 Code | | || ICC | 0.74 | | | 0.74 | | || N | 37 Code | | | 35 Code | | || Observations | 42872 | | | 39639 | | |
| Marginal R2 / Conditional R2 | 0.027 / 0.746 | | | 0.118 / 0.774 | | |

```
model_2a <- glmer(location ~ settings + num_of_peaks + (1|Code),data= total_air, family = binomial(link="logit"), na.action = na.omit)

model_2b <- glmer(location ~ settings + num_of_peaks + (1|Code),data= total_skin, family = binomial(link="logit"), na.action = na.omit)

tab_model(model_2a, model_2b, digits= 3, title= "Mixed effect models for Location ~ Setting + Number of peaks + random(Participant) for number of peaks data for Air and Skin sensor")
```

Mixed effect models for Location ~ Setting + Number of peaks + random(Participant) for number of peaks data for Air and Skin sensor

|  | location | | | location | | |
| Predictors | Odds Ratios | CI | p | Odds Ratios | CI | p |
| (Intercept) | 0.240 | 0.043 – 1.339 | 0.104 | 0.161 | 0.025 – 1.026 | 0.053 |
| settings [Urban] | 1.157 | 0.306 – 4.376 | 0.830 | 2.379 | 0.522 – 10.831 | 0.263 |
| num\_of\_peaks | 3.418 | 1.244 – 9.390 | **0.017** | 3.617 | 1.379 – 9.486 | **0.009** |
| SD (Intercept) | 1.000 |  |  | 1.000 |  |  |
| SD (Observations) | 2.718 |  |  | 2.718 |  |  |
| Random Effects | | | | | | |
| σ2 | 3.29 | | | 3.29 | | || τ00 | 0.00 Code | | | 0.00 Code | | || N | 28 Code | | | 34 Code | | || Observations | 52 | | | 57 | | |
| Marginal R2 / Conditional R2 | 0.620 / NA | | | 0.611 / NA | | |

```
model_3a <- glmer(Location ~ logTemperature + settings + logTemperature*settings + (1|Code),data= TNUMAIR_l, family = binomial(link="logit"))

model_3b <- glmer(Location ~ logTemperature + settings + logTemperature*settings + (1|Code),data= TNUMSKIN_l, family = binomial(link="logit"))

tab_model(model_3a,model_3b, digits = 3, title = "Mixed effect models for Location ~ Temperature + setting + interaction Temperature:setting + random(Participant) for raw data Air and Skin sensor")
```

Mixed effect models for Location ~ Temperature + setting + interaction Temperature:setting + random(Participant) for raw data Air and Skin sensor

|  | Location | | | Location | | |
| Predictors | Odds Ratios | CI | p | Odds Ratios | CI | p |
| (Intercept) | 54.228 | 18.432 – 159.544 | **<0.001** | 79.163 | 25.792 – 242.974 | **<0.001** |
| logTemperature | 0.766 | 0.722 – 0.813 | **<0.001** | 2.616 | 2.471 – 2.769 | **<0.001** |
| settings [Urban] | 3.857 | 3.470 – 4.287 | **<0.001** | 2.645 | 2.401 – 2.913 | **<0.001** |
| logTemperature \* settings [Urban] | 0.652 | 0.589 – 0.721 | **<0.001** | 0.793 | 0.727 – 0.865 | **<0.001** |
| SD (Intercept) | 22.556 |  |  | 22.852 |  |  |
| SD (Observations) | 2.718 |  |  | 2.718 |  |  |
| Random Effects | | | | | | |
| σ2 | 3.29 | | | 3.29 | | || τ00 | 9.71 Code | | | 9.79 Code | | || ICC | 0.75 | | | 0.75 | | || N | 37 Code | | | 35 Code | | || Observations | 42872 | | | 39639 | | |
| Marginal R2 / Conditional R2 | 0.040 / 0.757 | | | 0.066 / 0.765 | | |

```
model_tempU <- glmer(Location ~ logTemperature + (1|Code),data= TNUMAIR_l[TNUMAIR_l$settings=="Urban",], family = binomial(link="logit"))

model_tempR <- glmer(Location ~ logTemperature + (1|Code),data= TNUMAIR_l[TNUMAIR_l$settings=="Rural",], family = binomial(link="logit"))

model_tempUS <- glmer(Location ~ logTemperature + (1|Code),data= TNUMSKIN_l[TNUMSKIN_l$settings=="Urban",], family = binomial(link="logit"))

model_tempRS <- glmer(Location ~ logTemperature + (1|Code),data= TNUMSKIN_l[TNUMSKIN_l$settings=="Rural",], family = binomial(link="logit"))

tab_model(model_tempU, model_tempR, model_tempUS, model_tempRS, digits = 3, title = "Mixed effect models for Location ~ Temperature + random(Participant) per Sensor(Air/Skin) and per setting(Urban/Rural)")
```

Mixed effect models for Location ~ Temperature + random(Participant) per Sensor(Air/Skin) and per setting(Urban/Rural)

|  | Location | | | Location | | | Location | | | Location | | |
| Predictors | Odds Ratios | CI | p | Odds Ratios | CI | p | Odds Ratios | CI | p | Odds Ratios | CI | p |
| (Intercept) | 1689.470 | 178.985 – 15947.149 | **<0.001** | 9.025 | 4.017 – 20.275 | **<0.001** | 7409.411 | 48.060 – 1142299.825 | **0.001** | 12.310 | 4.660 – 32.514 | **<0.001** |
| logTemperature | 0.422 | 0.384 – 0.464 | **<0.001** | 0.518 | 0.482 – 0.556 | **<0.001** | 1.846 | 1.713 – 1.989 | **<0.001** | 3.125 | 2.930 – 3.334 | **<0.001** |
| SD (Intercept) | 96.232 |  |  | 4.736 |  |  | 290.736 |  |  | 6.550 |  |  |
| SD (Observations) | 2.718 |  |  | 2.718 |  |  | 2.718 |  |  | 2.718 |  |  |
| Random Effects | | | | | | | | | | | | |
| σ2 | 3.29 | | | 3.29 | | | 3.29 | | | 3.29 | | || τ00 | 20.86 Code | | | 2.42 Code | | | 32.18 Code | | | 3.53 Code | | || ICC | 0.86 | | | 0.42 | | | 0.91 | | | 0.52 | | || N | 36 Code | | | 15 Code | | | 32 Code | | | 15 Code | | || Observations | 31527 | | | 11345 | | | 27148 | | | 12491 | | |
| Marginal R2 / Conditional R2 | 0.024 / 0.867 | | | 0.086 / 0.473 | | | 0.010 / 0.908 | | | 0.169 / 0.599 | | |
